# RNF20/RNF40 supports the aggressive behavior in cervical cancer by regulating a peroxisome-based anti-ferroptotic mechanism

**DOI:** 10.1186/s12964-025-02279-9

**Published:** 2025-07-01

**Authors:** Shaishavi Jansari, Anna Blandau, Evangelos Prokakis, Daniela Grimm, Anja Maria Naßl, Stefan Küffer, Wiebke Möbius, Christian Dullin, Fernanda Ramos-Gomes, Leona Schüürhuis, Lena Fritsche, Laura Schridde, Matthias Plessner, Carolin. A. Bast, Fabian A. Gayer, Sven Thoms, Julia Gallwas, Florian Wegwitz

**Affiliations:** 1 Molecular Gynecology, Department of Gynecology and Obstetrics, University Medical Centre Göttingen, Robert-Koch-Straße 40, 37075 Göttingen, Germany; 2 Department of Urology, Medical Faculty, University Hospital Düsseldorf, Heinrich Heine University Düsseldorf, Düsseldorf, Germany; 3 Institute of Pathology, University Medical Centre Göttingen, Göttingen, Germany; 4 Department of Neurogenetics (Electron Microscopy), Max-Planck-Institute for Multidisciplinary Sciences (City Campus), Göttingen, Germany; 5 Translational Molecular Imaging, Max-Planck-Institute for Multidisciplinary Sciences (City Campus), Göttingen, Germany; 6 Clinical and Interventional Radiology, University Medical Center Göttingen, Göttingen, Germany; 7 Department of Diagnostic and Interventional Radiology, University Hospital Heidelberg, Heidelberg, Germany; 8 Department of Biochemistry and Molecular Medicine, Medical School OWL, Bielefeld University, Bielefeld, Germany; 9 Department of Urology, University Medical Centre Göttingen, Göttingen, Germany; 10 Department of Child and Adolescent Health, University Medical Centre Göttingen, Göttingen, Germany

**Keywords:** Cervical cancer, RNF20/RNF40, H2Bub1, Peroxisomes, Ferroptosis

## Abstract

**Background:**

Cervical cancer is the fourth most common cancer entity in women worldwide. Currently, malignant lesions are clinically managed by surgery, conventional chemotherapy, and/or radiotherapy. However, a significant fraction of patients with cervical cancer does not respond to such treatments, highlighting the need for personalized targeted therapies. Histone 2B monoubiquitination (H2Bub1) is an epigenetic marker catalyzed by the RNF20 and RNF40 heterodimeric E3 ligase complex and is strongly involved in the regulation of gene expression. Despite its well-established significance in various malignant diseases, the role of RNF20 and RNF40 in cervical cancer remains poorly understood.

**Methods:**

We investigated the role of RNF20 and RNF40 in cervical cancer cells by leveraging paraffin-embedded IHC staining on patient material, in vitro functional assays, in vivo CAM assays, flow cytometry, various microscopy-based techniques, ChIP-qPCR, as well as genome-wide transcriptome analysis from cell lines and publicly available datasets.

**Results:**

We showed that high RNF20 and RNF40 levels correlate with cervical cancer cell aggressiveness and poor patient prognosis. In addition, pathway enrichment analyses identified that the RNF20/RNF40/H2Bub1-axis positively regulates the peroxisome function. Peroxisomes play a key role in lipid metabolism and the homeostasis of reactive oxygen species. Loss of RNF20 and RNF40 leads to downregulation of peroxisome-related genes such as *PRDX5*,* PEX6*, and *PMVK*, and thus impaired peroxisomal biogenesis, ROS metabolism, and increased lipid peroxidation, ultimately resulting in ferroptotic programmed cell death induction.

**Conclusion:**

Together, our results indicate that interfering with RNF20 and RNF40 driven H2Bub1 and the peroxisome transcriptional program could provide a novel target for a therapeutic approach against aggressive cervical cancer.

**Supplementary Information:**

The online version contains supplementary material available at 10.1186/s12964-025-02279-9.

## Background

Cervical cancer (CC) is the fourth most common cancer entity in women across the globe with 662 301 new cases and 348 874 deaths reported in 2022 [1]. In 90% of cases, CC is caused by persistent Human Papillomavirus (HPV) infection. This persistent HPV infection results in cervical intraepithelial neoplasia (CIN) which later progresses into invasive CC. CC is histo-pathologically classified into squamous cell carcinoma, adenocarcinoma, and mixed or adenosarcoma [2]. Primary preventive measures for CC include bivalent, quadrivalent, and nonavelent vaccination against the high-risk HPV types 16, 18, and other serological subtypes; while secondary preventive measures include regular cytological screening and testing for HPV infection [3]. Surgical removal of the cancerous tissue represents the most common approach to treat early-stage CC by total and radical hysterectomy, loop electrosurgical excision procedure (LEEP), or trachelectomy [4]. At advanced stages, especially in the context of recurrent and/or metastatic diseases, CC has a very poor prognosis [5, 6]. Patients suffering from such aggressive forms of this malignancy are commonly treated with conventional chemo- or chemoradiotherapy and palliative care [7]. Standard first-line therapies combine platinum-based drugs, anti-angiogenic drugs, and immune checkpoint inhibitors; but further compounds like topotecan, paclitaxel, and other non-platinum compounds like 5-fluorouracil and bleomycin are employed in some cases [8]. However, such treatments often lose efficiency over time, highlighting the need to establish novel therapeutic strategies to improve CC patient outcomes.

Initiation and progression of CC has been closely linked to abnormal regulation of oxidative stress and programmed cell death [9]. For instance, chronic HPV infection has been shown to induce mitochondrial stress, leading to aberrantly high reactive oxygen species (ROS) levels and leading to the induction of apoptotic or autophagic signaling [10, 11]. Another alternate programmed cell death pathway involving an iron (Fe^2+^)-dependent generation of lipid peroxides as well as free radicals is ferroptosis [12]. The oncogene-induced elevated ROS levels render cancer cells particularly prone to targeted ferroptosis induction compared to normal cells [13, 14]. This non-apoptotic form of cell death relies on ROS-dependent peroxidation of polyunsaturated fatty acids (PUFA), ultimately leading to the disruption of membrane integrity. Consequently, crucial factors like the System X_c_^−^ (cysteine/glutamate antiporter), GPX4, or FSP1 protects against elevated lipid-ROS accumulation and ferroptosis induction as described in various cancer entities [15]. Peroxisomes are organelles that are deeply involved in the metabolic reprogramming of cancer cells, as they regulate lipid metabolism, long-chain fatty acid oxidation, and ROS metabolism, thereby promoting tumorigenesis by fulfilling the energy needs of cancer cells [16]. Recent studies have shown that the dysregulated peroxisome homeostasis, ether lipid synthesis, and ROS scavenging functions are implicated in increased susceptibility to ferroptosis [17, 18]. However, the precise function of peroxisomes in ferroptosis regulation remains insufficiently understood.

Histones undergo various post-translational modifications (PTMs) like methylation, acetylation, phosphorylation, ubiquitination, ADP ribosylation, and SUMOylation, generating a territory for precise regulation of gene expression through chromatin accessibility modulation and/or recruitment of various factors [19–21]. Because these PTMs tightly control chromatin compaction levels, they also deeply influence further crucial cellular processes like DNA replication, DNA damage/repair, or mitotic fidelity [22–24]. Interestingly, dysregulation of epigenetic factors that modulate these PTMs, known as writers, erasers, and readers, is frequently observed and implicated in cancers [25–27]. Hence, there have been significant research efforts in the past two decades to develop anti-cancer therapeutics targeting epigenetic factors [28].

Monoubiquitination of the H2B subunit of histones at lysine 120 residue (H2Bub1) is catalyzed by the heterodimeric E3 ligase complex of RING finger proteins RNF20 and RNF40 [29, 30]. H2Bub1 regulates gene expression by favoring transcription elongation of RNA polymerase II at specific genes through histone crosstalk with H3K4me3 and H3K79me3 [31, 32]. Further, H2Bub1 also favors DNA damage repair mechanisms through changes in chromatin accessibility and recruitment of factors involved in DNA repair [33, 34]. In normal cells, H2Bub1 governs the differentiation of stem cells by inducing lineage-specific gene activation [35, 36]. In cancer cells, H2Bub1 shows tumor-suppressive functions in inflammation-driven colorectal cancer [37, 38], lung adenocarcinoma [39], and ovarian cancer [40]. In contrast, tumor-supportive roles have been described in hepatocellular carcinoma [41], and MLL-rearranged leukemia [42]. In line with these results, our group recently demonstrated tumor-promoting functions of H2Bub1 in HER2-positive breast cancer (HER2^+^-BC) [43], colorectal [44], and triple-negative breast cancer (TNBC) [45] by supporting the actin cytoskeleton dynamics, anti-apoptotic signaling, and glycolytic capacity, respectively. However, the role of these RNF20/RNF40/H2Bub1-axis in CC remains unknown.

In the present study, we investigated the role of RNF20 and RNF40 in CC cells. Our work demonstrates a previously unknown tumor-supportive role of RNF20 and RNF40 by regulating the peroxisome functions and thereby suppressing ferroptosis. Together, our data establish the H2Bub1 driven by RNF20 and RNF40 as an attractive epigenetic pathway for the development of diagnostic and/or therapeutic strategies to treat advanced-stage CC.

## Methods

### Publicly available patient data

Publicly available mRNA-seq and patient survival data of normal cervical tissues and cervical cancer samples were retrieved from the TCGA-GTEx and TCGA-CESC datasets (source: xenabrowser.net). The CutoffFinder tool (v1, https://molpathoheidelberg.shinyapps.io/CutoffFinder_v1/) was used to determine the best appropriate *RNF20* or *RNF40* expression levels thresholds discriminating high- and low-expressing groups. The “H2Bub1-score” was calculated based on the average of *RNF20* and *RNF40* z-scores in the respective datasets.

### Single-cell sequencing analysis

The publicly available dataset with combined matrices of 5 adenocarcinomas or 3 squamous cell carcinomas was retrieved from GEO (GSE197461) and was analyzed using the Seurat package (v 5.0.1) in R v4.3.2. Cells with high content (over 5%) of mitochondrial RNA were filtered. The cells with less than 200 or more than 9000 genes sequenced were removed and the data was normalized and scaled. Linear dimensionality reduction (principal component analysis, PCA) was performed and Uniform manifold approximation and projection (UMAP) was run. Tumor clusters were identified using SingleR [46] and validated by visualizing the expression of *EPCAM* and *KRT18* for ADC; *CDKN2A* and *KRT14* for SCC. Within the tumor clusters, expression levels of *RNF20* and *RNF40* were correlated with *MKI67* expression. For this purpose, cells that did not show transcripts of either of the genes were filtered out.

### Immunohistochemical staining

2-µm tumor paraffin sections were immunostained with antibodies against RNF20 (1:200, EDTA pH6; Cell Signalling Technology), RNF40 (1:250, EDTA pH6; Abcam) and Ki67 (Dako, ready to use) according to a standard protocol on an Autostainer (Dako Agilent, USA). In brief, antigen retrieval was performed at 95 °C in pH 6 or pH 9 Envision FLEX target retrieval solution in a PT Link Module (Agilent, USA), followed by 1 h incubation with primary antibodies. Samples were washed with PBS and incubated with an appropriate secondary antibody (EnVision Flex+, Dako) for 30 min. Immunohistochemistry (IHC) pictures were acquired in a TIFF format with an IX-83 Olympus Microscope device. Pictures were opened in ImageJ (v1.54 m, release date: 5 December 2024) and converted to RGB format. Subsequent color deconvolution of H (hematoxylin) and D (DAB) was performed and the H channel was utilized as a region of interest (ROI) to mark the nuclei after threshold adjustment (upper = 0, lower = 150) and binary processing (median with rad = 3 and choosing the watershed option). Thereafter, the number of nuclei particles was retrieved using the following criteria: (a) size: 40-3000, (b) circularity: 0.3-1.0 and (c) edge exclusion. Thereafter, the mean intensity and area per ROI was calculated. Finally, the ROI overlay was transferred to the respective DAB channel, which was inverted in advance, and mean DAB intensity in each ROI was obtained. DAB mean intensity values were subsequently collected from all brightfield pictures [*n* = 2 fields per normal (adjacent) or tumor area)] for each marker and histo-score (H-score) calculation was performed using Microsoft^®^ Excel 2016, relying on the recent H-score quantification guidelines [47] Finally, the resulting findings were plotted using Graphpad Prism (v.8.0.1).

### Cell culture, transfections, and functional assays

HeLa (ATCC ^®^ CCL-2 ™) and SiHa (ATCC ^®^ HTB-35 ™) cells were purchased from the American Type Culture Collection (ATCC) and cultivated in Minimum Essential Medium (MEM) (Biowest) supplemented with 10% fetal bovine serum (FBS) (Biowest), 1x penicillin/streptomycin (Gibco) at 37 °C with 5% CO_2_. siRNA transfections were performed using DharmaFECT1 (Dharmacon) in OptiMEM GlutaMAX (Gibco) according to the manufacturer’s protocol. Proliferation kinetics, sensitization to erastin, and tumorsphere numbers were recorded using a Celigo ^®^ S imaging cytometer (Nexcelom Bioscience LLC). For endpoint analysis of proliferation kinetics, sensitization to Erastin, rescue with Ferrostatin-1, inhibition with CDK9 (CDK9i) and colonies for clonogenic assays, cells were fixed with methanol (J.T.Baker) for 20 min, stained with 0.25% crystal violet in 20% methanol (J.T.Baker) for 20 min, washed, air dried and scanned with an Epson Perfection V850 PRO. Details of used functional assay protocols and a list of siRNAs (Table S1) are provided in the supplementary data. The results from the analysis of functional assays were plotted using GraphPad Prism v8.0.1.

### RNA isolation and real-time quantitative PCR (RT-qPCR)

RNA isolation, cDNA synthesis, and RT-qPCR were performed as previously described [48]. The primer sequences used in this study are provided in Table S2. Detailed protocols of RNA extraction and cDNA synthesis are provided in the Supplementary Data. Results were plotted using GraphPad Prism v8.0.1.

### mRNA library Preparation and data analysis

mRNA sequencing (mRNA-seq) library was performed as described previously [43] 48 h after transfection using the TruSeq ^®^ RNA Library Prep Kit v2 (Illumina) according to the manufacturer’s instructions. RNF40 Knockdown (KD) samples were sequenced (single-end 50 bp) on a HiSeq4000 (Illumina) at the NGS Integrative Genomics Core Unit (NIG) of the University Medical Center Göttingen (UMG). RNF20 KD samples were sequenced (paired-end 100 bp) on a DNBSEQ-G400 (BGI Genomics). mRNA-seq data were then processed and analyzed in the Galaxy environment provided by the “Gesellschaft für Wissenschaftliche Datenverarbeitung GmbH Göttingen” (GWDG). Briefly, the first 11 nucleotides of the raw reads were trimmed (FASTQ Trimmer, version 1.0.0). Human mRNA-seq data were aligned to the hg38 reference genome using the TopHat Gapped-read mapper (version 2.1.1) [49]. Read counts per gene were calculated with featureCounts (version 2.0.1 + galaxy2). Finally, differential gene expression analysis and normalized counts were obtained using DESeq2 (version 2.11.40.6 + galaxy2) [50]. To identify differentially regulated genes upon RNF40 and RNF20 loss, we used a cut-off of|log2 fold change| ≥0.5; *p*-val < 0.05 and basemean ≥ 15. Pathway enrichment analysis was performed using the Gene Set Enrichment Analysis (GSEA, v4.1.0, source: https://www.gsea-msigdb.org/gsea/msigdb).

### Protein analysis

Protein extraction and quantification were performed according to the standard protocols of the lab [43]. Samples were subsequently analyzed by Western Blot. Detailed descriptions are provided in the supplements. Full and uncropped western blots are available in the supplemental data (Supplementary Material 7).

### Chromatin Immunoprecipitation (ChIP)

Chromatin immunoprecipitation was performed as described previously [43] 72 h after siRNA-mediated transfection using antibodies against H2Bub1 (Cat. No. 5546 S, Cell Signaling Technology) followed by RT-qPCR.

### Cellular ROS measurement

HeLa cells (12-well plates) were treated with the siRNA of interest as indicated before. 72 h after transfection, 5 µM CellROX^®^ green reagent (#C10444, Thermo Fisher) was added to the cell culture medium and incubated for 30 min at 37 °C. Excess dye was removed by washing the cells thrice with PBS. Cells were trypsinized and resuspended in PBS containing 1% FBS. The amount of cellular ROS levels was measured using the FITC channel by flow cytometry analysis (CytoFlex, Beckman Coulter). Results were analyzed with FlowJo (v10.8.1) and plotted using GraphPad Prism v8.0.1.

### Lipid peroxidation measurement

HeLa and SiHa cells (12-well plates) were treated with siRNA or inhibitors as indicated. 72 h after treatment, 1 µM BODIPY-C11 reagent (#27086, Cayman Chemicals) was added to the culture medium and incubated for 2–3 h at 37 °C. Excess dye was removed by washing the cells twice with PBS. Cells were trypsinized and resuspended in PBS containing 1% FBS. Flow cytometry (CytoFlex, Beckman Coulter) measurement was utilized to measure oxidized BODIPY-C11 dye (FITC channel), a parameter directly proportional to the amount of lipid peroxidation. Assessment of the PE channel fluorescence was used as an internal control for dye incorporation. Results were analyzed with FlowJo (v10.8.1) and plotted using GraphPad Prism v8.0.1.

### Immunofluorescence staining

The cells were treated with siRNA, washed with PBS 72 h post-transfection, fixed in 4% PFA for 10 min, and later stored in 0.01% PFA in PBS. Nuclei were stained with DAPI. Antibodies against PMP70 (AF488, green) and PEX14 (10594-1-AP, green) stain the peroxisomal membrane. For PMP70 staining, full confocal z-stacks were acquired with a step size of 160 nm using a laser scanning microscope (Zeiss LSM900). A pixel size of 60 nm was achieved with a 63 × 1.4 NA objective. Images were analyzed using a custom Cell Profiler pipeline. In brief, DAPI-defined nuclei propagate cell bodies for single-cell analysis. Then, a top-hat filter and thresholding were applied to the PMP70 signal to define peroxisomal objects, which were related to parent cells. The data were analyzed by quantification of the number and mean area of peroxisomal objects per parent cell.

### STED microscopy

High resolution images of HeLa cells were acquired using a STEDYCON STED microscope (Abberior Instruments) equipped with an UPlanSApo 100×/1.45 Oil [infinity]/0.17/FN26.5 objective. PEX14 was excited at 640 nm wavelength, and STED was performed using a pulsed depletion laser at 775 nm wavelength with gating of 1 to 7 ns and dwell times of 5 to 10 µs. Pixel sizes of 40 nm were used for STED nanoscopy, and each line was scanned 5 times (line accumulations). The pinhole was set to 60 μm. DAPI was excited at 405 nm wavelength and was acquired in the confocal mode. Signals were detected using avalanche photodiodes (APD). Images were acquired by a browser-based graphical user interface named “STEDYCON smart control” STED microscopy software (Abberior Instruments). The images were analyzed using ImageJ and quantification was done using GraphPad Prism v8.0.1. Detailed protocols for fixation of HeLa cells and IF staining are available in the supplemental data.

### Electron microscopy

#### For mitochondria

HeLa cells were transfected in 10 cm culture dishes; 72 h after transfection the cells were fixed in culture dishes with 4% formaldehyde and 2.5% glutaraldehyde in 0.1 M phosphate buffer pH 7.4; followed by post fixation with 1% OsO_4_ (Science Services, Germany) in 0.1 M phosphate buffer at 4 °C and embedded in Epon (Serva, Germany) after dehydration with ethanol and en bloc staining with 1.5% uranyl acetate (Merck)/ 1.5% tungstophosphoric acid (Merck, Germany) in 70% ethanol. Ultrathin sections of cultured cells were cut parallel to the substrate using an UC7 Ultramicrotome (Leica, Germany) and stained with UranylLess^®^ (Science Services, Germany). Sections were analyzed with a LEO EM912 Omega (Zeiss, Germany) and digital micrographs were obtained with an on-axis 2048 × 2048 CCD camera (TRS).

#### For peroxisomes

HeLa cells were transfected in 10 cm petridish and 72 h after transfection, the cells were fixed in culture dishes with 4% formaldehyde and 0.25% glutaraldehyde in 0.1 M phosphate buffer pH 7.4 and infiltrated overnight in 2.3 M sucrose in 0.1 M phosphate buffer pH7, mounted on aluminum pins and frozen in liquid nitrogen. Ultrathin cryosections were prepared using a diamond knife (cryoimmuno, 35°, Diatome, Biel Switzerland) and a UC6 ultramicrotome equipped for cryosectioning (Leica Microsystems, Vienna, Austria). Ultrathin sections were placed on hexagonal 100 mesh copper grids (Science Services, Munich, Germany) and labeled for Catalase (Rockland, Limerick, USA), followed by incubation with protein A-gold (10 nm, Cell Microscopy Core, UMC Utrecht, The Netherlands). Images were taken with EM900 transmission electron microscope (Zeiss Microscopy GmbH, Oberkochen, Germany) using an on-axis 2k CCD camera (TRS, Moorenweis, Germany).

### Chorioallantoic membrane (CAM) assay

CAM assays were performed as described previously [45]. Briefly, HeLa cells treated with DMSO, CDK9i (1µM) and Erastin (20µM) for 48 h, were trypsinized and resuspended in Matrigel: MEM (1:1). For every replicate, 4 × 10^6^ cells in 35 µl were implanted onto a 10-day-old chicken embryo CAM and incubated for an additional 7 days. The isolated tumors were fixed and embedded in paraffin (Detailed protocol in supplemental data). FFPE-CAM specimens were imaged using an in vivo microCT scanner (QuantumGX2, Revvity) with the following parameters: tube voltage of 70 kV, tube current of 110 µA, a 25 × 25 mm² field of view, a 0.5 mm aluminum filter, and a total acquisition time of 4 min. The raw data were reconstructed into a 1024 × 1024 × 1024 voxel matrix, yielding an isotropic voxel size of 24.4 μm. Tumor volume measurements were performed using Scry, a 3D rendering and analysis software developed by C. Dullin. Threshold-based segmentation was used to differentiate PTA-stained tissue from surrounding paraffin, and a 3D cutting tool was applied manually to remove residual CAM tissue from the tumor. Analyzed results were plotted using GraphPad Prism v8.0.1.

## Results

### High RNF20 and RNF40 levels lead to poor outcomes in cervical cancer patients

**Fig. 1:**
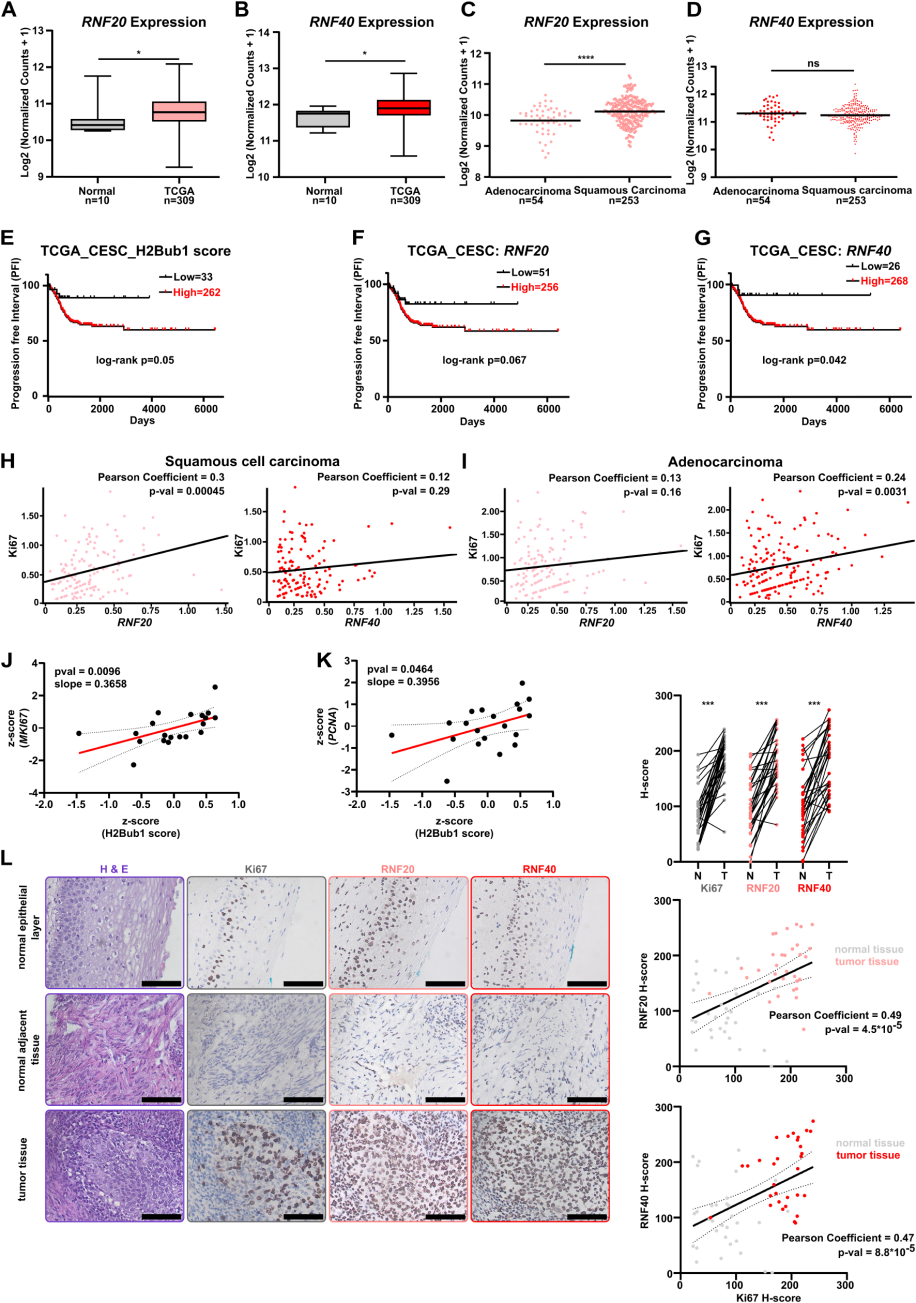
High RNF20 and RNF40 leads to poor prognosis of cervical cancer patients: **A** and **B**: Comparison of healthy and malignant cervical tissues showing higher *RNF20* (**A**) and *RNF40* (**B**) expression in the tumor tissues. Datasets: Genotype Tissue Expression (GTEx) and The Cancer Genome Atlas for Cervical Squamous Cell Carcinoma and Endocervical Adenocarcinoma (TCGA-CESC) respectively; data retrieved from https://xenabrowser.net/. **C and D**: Comparison of *RNF20* and *RNF40* expression in adenocarcinoma (**C**) and squamous (**D**) CC (TCGA-CESC). **E to G**: Impact of the H2Bub1 score, the *RNF20* and *RNF40* expression on progression-free interval (PFI) of CC patients. (PFI: H2Bub1 score cut-off: − 0.905) and squamous carcinoma patients (PFI: H2Bub1 score cut-off: − 1.09). **H**: Correlation of the proliferation marker *MKI67* expression with *RNF20* (left panel) or *RNF40* (right panel) in SCC cells. **I**: Correlation of the proliferation marker *MKI67* expression with *RNF20* (left panel) or *RNF40* (right panel) in ACC cells. **J and K**: Correlation of the proliferation marker *MKI67* expression (**J**) and *PCNA* expression (**K**) with H2Bub1 score from CC cell lines from DepMap portal. (source: https://depmap.org/portal/). **L**: Hematoxylin and Eosin staining (H & E) and immunohistochemical detection of Ki67, RNF20 and RNF40 on *n* = 32 CC biopsies with normal epithelial lining, normal adjacent and tumor areas (left panel). In addition, respective quantifications (right panel) were performed using the histo-score method [47]. Black scale bar: 10 μm. Statistical test: **A-D**: Mann-Whitney test, **E-G**: Log-rank test, **L**: Upper graph (normal versus tumor tissue): for Ki67 and RNF40, Wilcoxon matched-pairs signed rank test was applied, while for RNF20, paired t-test was applied. Middle and Lower graph (Ki67 to RNF20 or RNF40 correlation): Pearson correlation analysis was performed; *p-val < 0.05, *** *p* < 0.005, ****p-val ≤ 0.0001, *p*-val > 0.05 = not significant

In recent work, we reported tumor-supportive functions of H2Bub1 in HER2^+^-BC [43] and TNBC [45] however, the role of this histone PTM is still elusive in CC. To fill this gap and investigate the potential association of RNF20 and RNF40 with disease development and outcome, we explored the publicly available patient mRNA-Seq dataset “Genotype-Tissue Expression” (GTEx) and *Cell Carcinoma and Endocervical Adenocarcinoma* from *The Cancer Genome Atlas* (TCGA-CESC). Here, we observed an increased *RNF20* and *RNF40* expression in CC lesions as compared to normal cervical tissues (Fig. 1A, B). *RNF20* expression levels were found elevated in squamous CC, whereas no difference in *RNF40* expression levels was identified between both squamous and adenocarcinoma groups (Fig. 1C, D). To take possible reciprocal compensatory effects of the two E3 ligases into consideration, we combined *RNF20* and *RNF40* expression data (H2Bub1 score) better reflecting the activity of the H2Bub1 pathway in the tumor samples. Strikingly, high H2Bub1-score was associated with poor overall survival in CC (Fig. 1E), and in the squamous carcinoma subgroup of patients (Fig. S1A). *RNF20* expression only showed a tendency to associate with poor disease outcomes (Fig. 1F and S1B). Interestingly, increased expression of *RNF40* was associated with poor survival outcomes in CC patients as well as in the subgroup of squamous CC patients (Fig. 1G and S1C), respectively. The subgroup of 48 adenocarcinoma patients was unfortunately too small to make any conclusions regarding a potential prognostic value of these two factors (data not shown). Surprisingly, neither *RNF20-* nor *RNF40-*expression showed a correlation with clinical (Fig. S1D, E) and TNM staging (Fig. S1F-K) of CC patients. Next, we leveraged a publicly available single-cell sequencing dataset of cervical squamous cell carcinoma (SCC) and adenocarcinoma (ADC) (GSE197461). We established the cell cluster identity using the SingleR tool [46] and confirmed the identified tumor clusters based on the expression of *CDKN2A* and *KRT14* for SCC (Fig. S1L, S1M). Interestingly, a positive correlation of *RNF20* but not *RNF40* to *Ki67* was observed in the tumor cell cluster of SCC, pointing at a potential oncogenic function of this RNF20 and RNF40 driven H2Bub1 (Fig. 1H). In a similar approach, we identified the cell cluster of ADC and confirmed the tumor cells cluster based on the expression of *EPCAM* and *KRT18* (Fig. S1N, S1O). Here, a positive correlation between *Ki67* and *RNF40* but not *RNF20* expression was observed (Fig. 1I), supporting an implication of RNF20 and RNF40 in CC aggressiveness. Further analysis of mRNA expression data retrieved from the DepMap platform confirmed our previous observations and showed a positive correlation between the H2Bub1 score and *MKi67* or *PCNA* in cervical cancer (CC) cell lines. (Figure 1J and K). Finally, we leveraged immunohistochemical (IHC) staining to visualize the expression of *Ki67*, *RNF20*, and *RNF40* in paraffin-embedded CC patient samples. As suggested by the previous results, tumor tissues expressed higher levels of *RNF20* and *RNF40* than normal and surrounding tissues (Fig. 1L). Furthermore, *RNF20* and *RNF40* levels correlated with *Ki67* levels. (Fig. 1L). Overall, analysis of both our own and publicly available patient data strongly suggests an implication of RNF20 and RNF40 in aggressive CC disease.

### H2Bub1 imparts tumor-supportive functions in cervical cancer

**Fig. 2:**
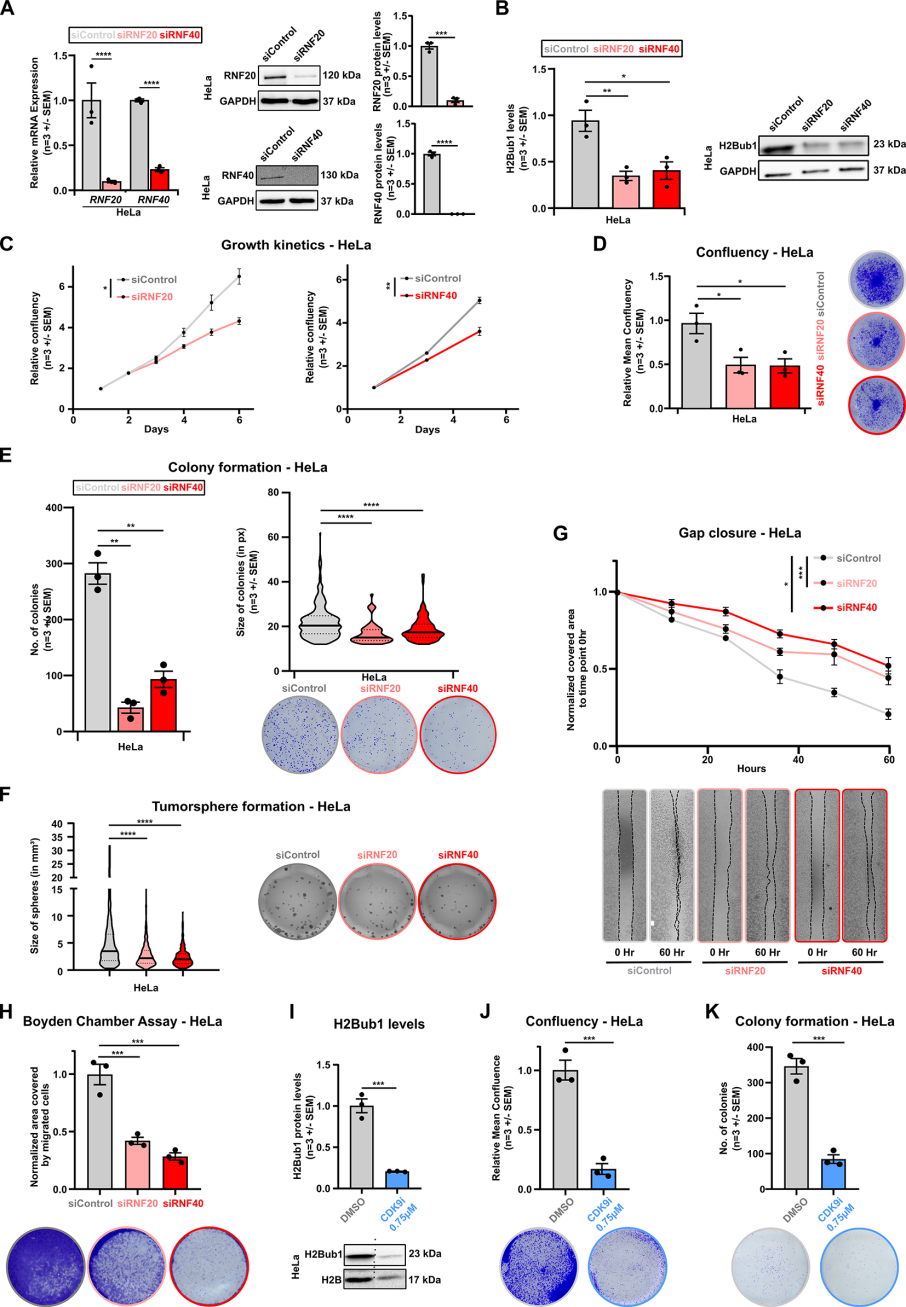
*RNF20* and *RNF40* plays a tumorigenic role in vitro: **A**: Real-time quantitative PCR (RT-qPCR) (Left panel) and Western blots along with quantification (Right panel) of *RNF20* and *RNF40* in siControl, siRNF20 and siRNF40-treated HeLa cells at 72 h of silencing. **B**: Western Blot of H2Bub1 in siControl, siRNF20, and siRNF40-treated HeLa cells at 72 h of silencing. Quantification is shown as a bar graph (left panel). **C– H**: Proliferation kinetics (**C**,** D**), colony (**E**), tumorsphere formation assay (**F**), Gap closure assay (**G**) and Boyden chamber assay (End point at 48 h post-seeding in inserts) (**H**) in siControl, siRNF20 and siRNF40 treated HeLa cells. **I**: Western blot of H2Bub1 in CDK9 treated (CDK9i) (0.75µM) HeLa cells 48 h after treatment (Top panel). Quantification as bar graph (Bottom panel). **J-K**: Relative Mean confluency at end-point (**J**) and colony formation assay (**K**) in CDK9i treated HeLa cells. Statistical test: **A**,** B**,** D**,** E**,** F**,** H**,** I**,** J**,** K**: Student *t*-test, **C**,** G**: AUC followed by Student t-test; *p-val < 0.05, **p-val ≤ 0.01, ***p-val ≤ 0.001, ****p-val ≤ 0.0001. Error bars: Standard error of the mean (SEM). All experiments were performed in biological triplicates per condition

To better understand the relationship between *RNF20-* and *RNF40-*driven H2Bub1 levels and CC cell aggressiveness, we performed in vitro functional assays for various oncogenic properties. Two cell lines were selected: the HeLa derived from ADC and the SiHa derived from SCC [51, 52]. We leveraged smart-pool and single siRNA-mediated KD to interfere with the *RNF20* and *RNF40* expression (Table S1). The efficiency of the KDs in HeLa (Fig. 2A) and SiHa (Fig. S2A) was validated via qRT-PCRs and western blot. Further, reduction of H2Bub1 levels as a consequence of RNF20 and RNF40 loss in HeLa and SiHa was verified with western blots (Fig. 2B, S2B). As previously observed in TNBC and HER2^+^-BC [43, 45], loss of RNF20 and RNF40 decreased the proliferation rate in HeLa (Fig. 2C, D) and SiHa (Fig. S2C, D) cells. The ability of individual tumor cells to form multicellular clusters under both adherent and non-adherent conditions has been linked with enhanced cancer stem cell properties. To examine whether the H2Bub1-signaling pathway influences these properties in CC cells, we conducted both colony and sphere formation assays. Interestingly, the ability of HeLa and SiHa cells to form colonies under 2D conditions and tumor spheres under non-adherent 3D conditions was greatly reduced in the absence of RNF20 and RNF40 (Fig. 2E-F, S2E-F). Subsequently, we studied the potential impact of RNF20 and RNF40 on the migratory properties of HeLa and SiHa cells (Fig. 2G-H, S2G-H). In line with our previous findings, a gap closure assay and Boyden chamber assay revealed that the loss of RNF20 and RNF40 both impaired tumor cell migration. To validate the functional involvement of H2Bub1 signaling in the tumorigenic potential of CC cells, we treated HeLa and SiHa cells with a CDK9 inhibitor (CDK9i, BAY-1251152) known to impair the ubiquitination on H2B efficiently [53] (Fig. 2I, S2I). Similar to the observations from siRNA-mediated KDs, we observed reduced proliferation rate (Fig. 2J, S2J) and clonogenic properties (Fig. 2K, S2K) upon CDK9i in Hela and SiHa cells.

We performed single siRNA-mediated KD of *RNF20* and *RNF40* and validated it via qRT-PCR in CC cell lines (Fig. S3A, S3B). The proliferation rate of CC cells was reduced upon single siRNA-mediated KD of *RNF20* and *RNF40* (Fig. S3C, S3D), showing similar results as smart pool. Together, our results establish a strong implication of RNF20 and RNF40 in the biology of CC cell lines in vitro and further support their association with patient survival.

### RNF20 and RNF40 regulates the expression of genes responsible for peroxisome functions

**Fig. 3:**
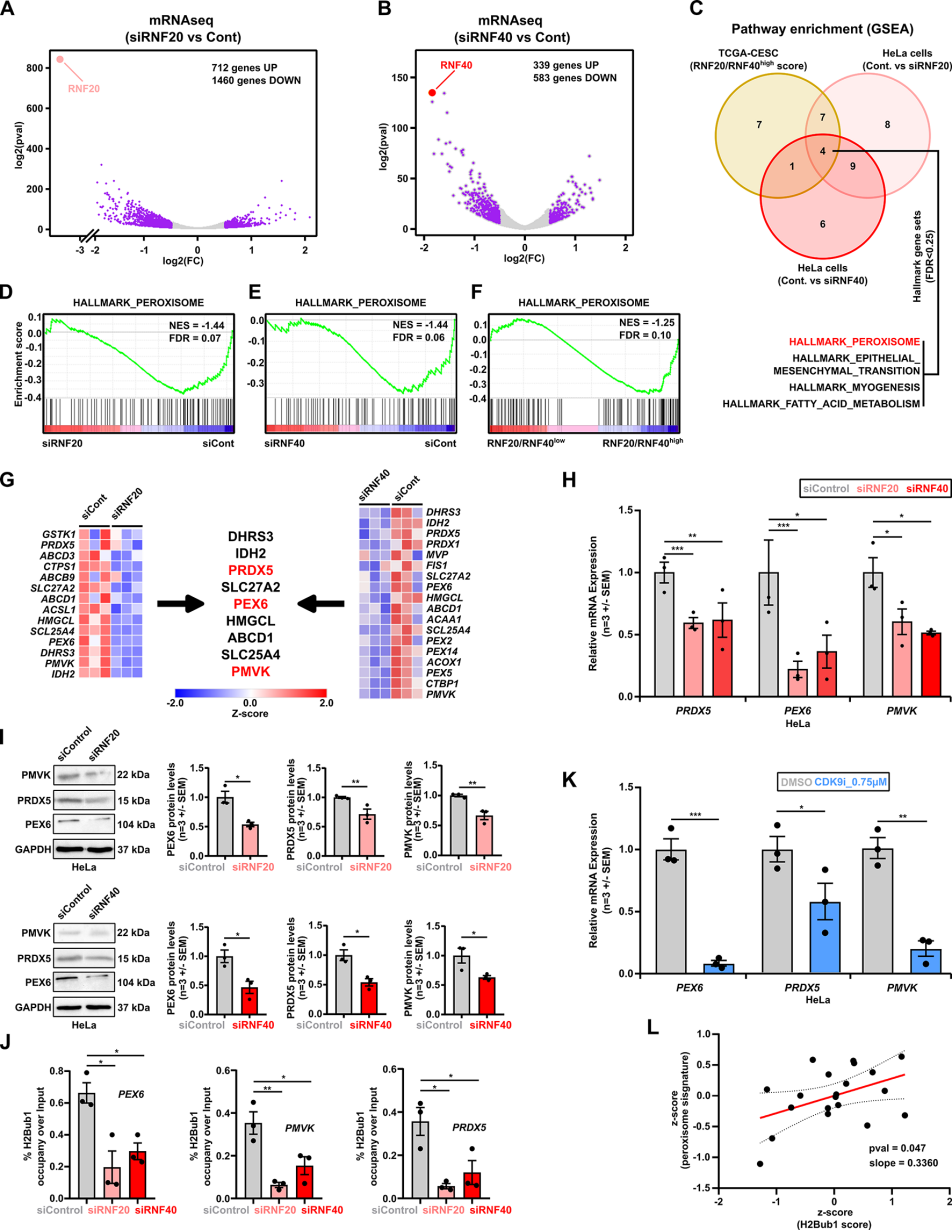
High *RNF20* and *RNF40* expression leads to upregulation of the “peroxisome gene signature”. **A and B**: mRNA-sequencing data identified gene expression changes occurring in HeLa cells 72 h after siRNF20 (**A**) and siRNF40 (**B**) treatment (|log2FC|>=0.5, pval < 0.05). **C**: GSEA identified gene sets commonly impaired upon RNF20 and RNF40 KD in HeLa cells that were at the same time enriched in RNF20/RNF40^high^ expressing cervical cancer patients (TCGA-CESC, https://portal.gdc.cancer.gov/). **D to F**: GSEA profiles of “HALLMARK_PEROXISOME” gene set significantly enriched in siControl treated HeLa cells (**D and E**) and RNF20/RNF40^high^ expressing cervical cancer patients (**F**). NES = Normalized Enrichment Score. FDR = False Discovery Rate. **G**: Commonly differentially regulated genes of “HALLMARK_PEROXISOME” in HeLa cells treated with either siRNF20 or siRNF40. **H-I**: RT-qPCR (**H**) and western blots (**I**) of peroxisome-related genes belongs to “HALLMARK_PEROXISOME” gene set, in siControl, siRNF20 and siRNF40 treated HeLa cells (72 h after KD). **J**: ChIP-qPCR of H2Bub1 occupied regions on *PEX6*,* PRDX5* and *PMVK* genes in siControl, siRNF20 and siRNF40 treated HeLa cells. Signal levels in control IgG samples: 0.0001–0.004% input for *PEX6*, 0.0001–0.0008% input for *PRDX5*, 0.0002–0.002% input for *PMVK.***K**: RT-qPCR of peroxisome-related genes in DMSO and CDK9i (0.75µM) treated HeLa cells (24 h of treatment). **L**: Correlation of peroxisome signature genes expression with H2Bub1 score from CC cell lines from DepMap portal. (source: https://depmap.org/portal/). Statistical test: **H-K**: Student *t*-test; *p-val < 0.05, **p-val ≤ 0.01, ***p-val ≤ 0.001. Standard error of the mean (SEM). All experiments were performed in biological triplicates per condition

To explore the H2Bub1-driven mechanisms underlying aggressive behavior in CC, we performed mRNA sequencing on HeLa cells treated with siRNF20 and siRNF40. Sequencing results showed a majority of genes downregulated (1460 and 583 upon siRNF20 and RNF40 treatment, respectively), whereas a small fraction of genes were upregulated (712 upon RNF20 KD and 339 upon RNF40 KD) (Fig. 3A-B). Interestingly, gene set enrichment analysis (GSEA) showed a reduction of the peroxisomal gene signature in HeLa cells upon *RNF20* or *RNF40* KD, but also in the TCGA-CESC patient cohort with concomitant low *RNF20*/*RNF40* expression score (Fig. 3C-F). Peroxisomes are organelles involved in lipid metabolism and oxidative stress management [54]. Lipid metabolism provides an energy source and helps meet the needs of proliferating cancer cells to generate cell membranes [55] whereas oxidative stress management supports cancer cells overcoming the excess ROS produced by aberrant metabolic activities [56]. Therefore, we hypothesized that the H2Bub1-mediated stimulation of the peroxisome transcriptional program might underlie the increased aggressiveness of *RNF20/RNF40* high-expressing CC tumors. To answer this question, we identified genes of the “peroxisome signature” that were commonly dependent on RNF20 and RNF40 in HeLa cells (Fig. 3G). Specifically, we found nine peroxisome-related genes significantly downregulated in siRNF20- and siRNF40-treated HeLa cells. We focused on three genes in particular (*PEX6*, *PRDX5*, and *PMVK*) because of their well-established critical roles in peroxisome homeostasis. *PEX6* encodes for an AAA-type ATPase involved in protein import into the peroxisome lumen [57, 58]. In addition, loss of PEX6 strongly impairs peroxisome biogenesis [59]. PRDX5 plays an important antioxidant function by scavenging hydrogen peroxides and alkyl hydroperoxides, while its loss results in enhanced oxidative stress [60]. *PMVK* encodes for a phosphomevalonate kinase involved in cholesterol precursor synthesis and its down-regulation impairs lipid metabolism [61]. The downregulation of *PEX6*, *PRDX5*, and *PMVK* upon RNF20 and RNF40 silencing was validated by RT-qPCR (Fig. 3H) and western blots (Fig. 3I). Downregulation of peroxisome-related genes upon RNF20 and RNF40 silencing via single siRNA-mediated KDs was also validated by RT-qPCR in CC cells to exclude eventual off-target effects (Fig. S3E, S3F). To confirm the association of the peroxisome signature impairment with the loss of RNF20/RNF40-H2Bub1 signaling, we performed chromatin immunoprecipitation and RT-qPCR (ChIP-qPCR) of H2Bub1 in siControl, siRNF20, and siRNF40-treated HeLa cells. As expected, KDs resulted in the loss of H2Bub1 occupancy at the gene body of the selected peroxisome-related genes *PEX6*, *PRDX5*, and *PMVK* (Fig. 3J). The loss of H2Bub1 upon CDK9i treatment also showed downregulation of peroxisome-related genes via RT-qPCR (Fig. 3K, S4A). Publicly available data of CC cell lines from the DepMap portal supported our previous observations and showed a significant positive correlation between the H2Bub1 score and the expression of peroxisome-related genes (Fig. 3L). Together, H2Bub1 driven by RNF20 and RNF40 supports peroxisome-related gene expression in CC cells.

### Peroxisome dysfunction results in the impairment of its ROS scavenging function in CC cells

**Fig. 4:**
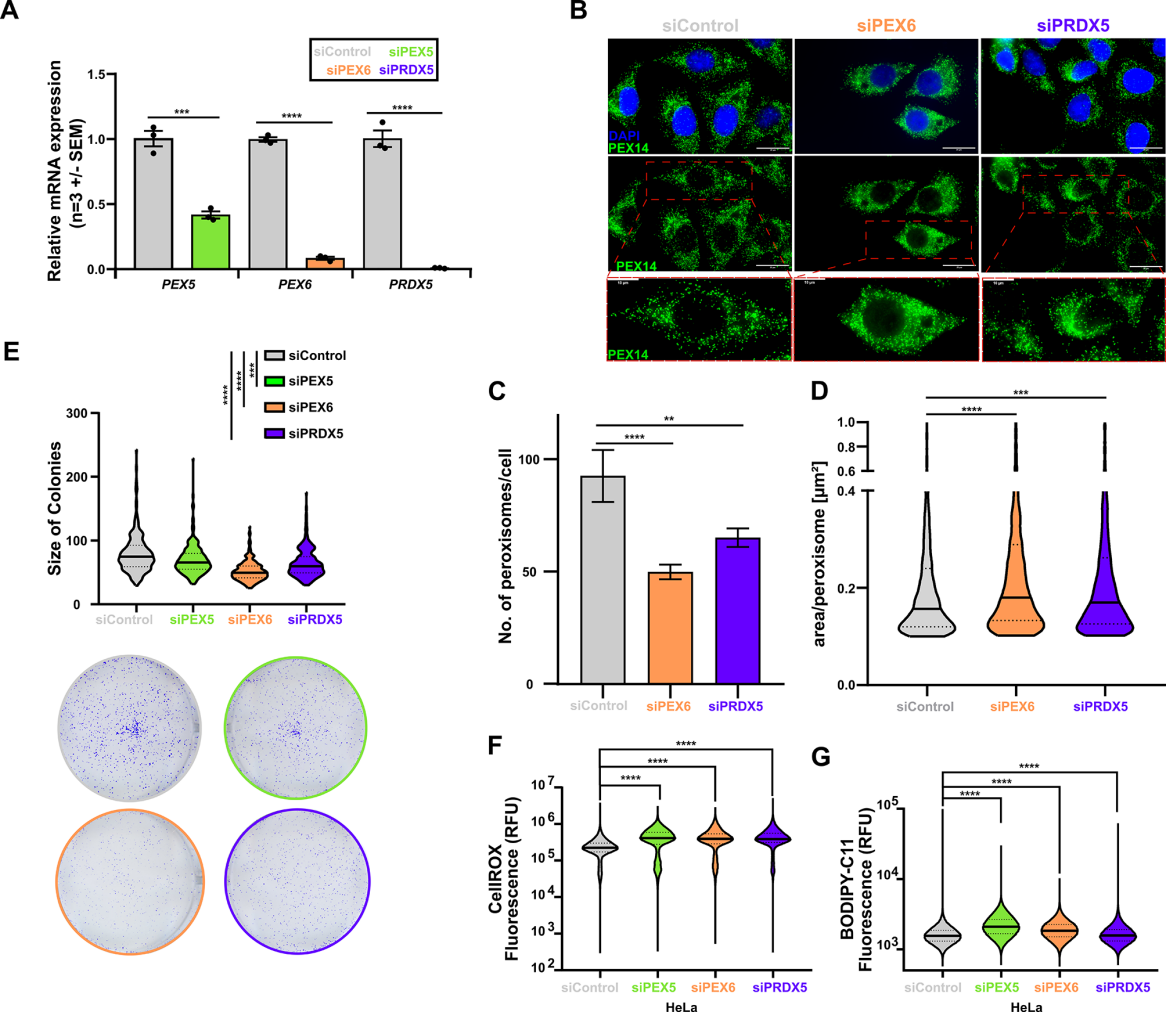
Peroxisome dysfunction leads to ROS imbalance and oxidative stress. **A**: RT-qPCR of *PEX5*, *PEX6* and *PRDX5* in siControl, siPEX5, siPEX6 and siPRDX5-treated HeLa cells at 72 h of silencing. **B-D**: Assessment of peroxisome distribution changes by PEX14 immunofluorescent staining (**B**) showing decreased peroxisome number (**C**) and increase in peroxisome size (**D**) for siControl, siPEX6 and siPRDX5 treated HeLa cells. Scale Bar: 10 μm. **E**: Colony formation assay in siControl, siPEX5, siPEX6 and siPRDX5-treated HeLa cells. **F-G**: Violin plots of ROS levels (**F**) and lipid peroxidation levels (**G**) in siControl, siPEX5, siPEX6 and siPRDX5-treated HeLa cells by CellROX and BODIPY-C11 staining respectively. Statistical test: **A**,** E**: Student *t*-test, **C**: One-way ANOVA, **D**: Kruskal-Wallis test, **F**,** G**: Mann-Whitney test; **p-val ≤ 0.01, ***p-val ≤ 0.001, ****p-val ≤ 0.0001. Error bars: Standard error of the mean (SEM). All experiments were performed in biological triplicates per condition

As H2Bub1 interference impaired the expression of several crucial genes of the peroxisomal function, we questioned if this observation could explain the reduced tumorigenic properties of CC cells upon RNF20/RNF40 loss. To answer this question, we downregulated *PEX6* and *PRDX5* and assessed changes in peroxisome properties. PEX5 is a shuttle receptor protein for matrix protein import from the cytosol into the peroxisome lumen [62]. siPEX5 was used as a positive control for interfering with peroxisome biogenesis [63, 64]. KD efficiency was validated with RT-qPCR (Fig. 4A). First, we assessed changes in peroxisome morphology by immunofluorescence (IF) staining of PEX14 or PMP70, two peroxisome membrane proteins. Interestingly and in a similar manner as PEX5 loss; PEX6 and PRDX5 KD led to a reduction of peroxisome number together with an increase of peroxisome size (Fig. 4B-D and Fig. S4B-D). To assess whether the impairment of peroxisomes governed through these RNF20- and RNF40-dependent genes, could affect the aggressiveness of CC cells, we performed a colony formation assay. Here, loss of PEX5, PEX6, and PRDX5 indeed impaired the clonogenic properties in HeLa cells (Fig. 4E). As peroxisomes play a crucial role in scavenging ROS, we investigated changes of ROS levels in PEX5-, PEX6- and PRDX5-silenced CC cells by flow cytometry. Interestingly, the KD of each of these genes strongly increased the intracellular ROS levels, a potential basis for decreased tumorigenic properties (Fig. 4F, S4E). Next to balancing cellular oxidative potential, peroxisomes are also involved in lipid biogenesis and metabolism. Interestingly, increased ROS levels can lead to an iron-dependent oxidation of membrane lipids, ultimately inducing ferroptosis [65]. Therefore, we hypothesized that an impairment of peroxisome function in CC tumor cells might ultimately activate ferroptosis. Indeed, by Bodipy-C11 staining, we observed that KD of *PEX5*, *PEX6*, and *PRDX5* dramatically increased the lipid peroxidation levels, a well-established marker of ferroptosis [66] in both HeLa (Fig. 4G, S4F) and SiHa (Fig. S4G) cells. Together, our results uncovered a so far unknown peroxisomal function dampening ferroptotic phenotypes in CC cells.

### Dysfunctional peroxisomes lead to ferroptosis in CC cells upon H2Bub1 loss

**Fig. 5:**
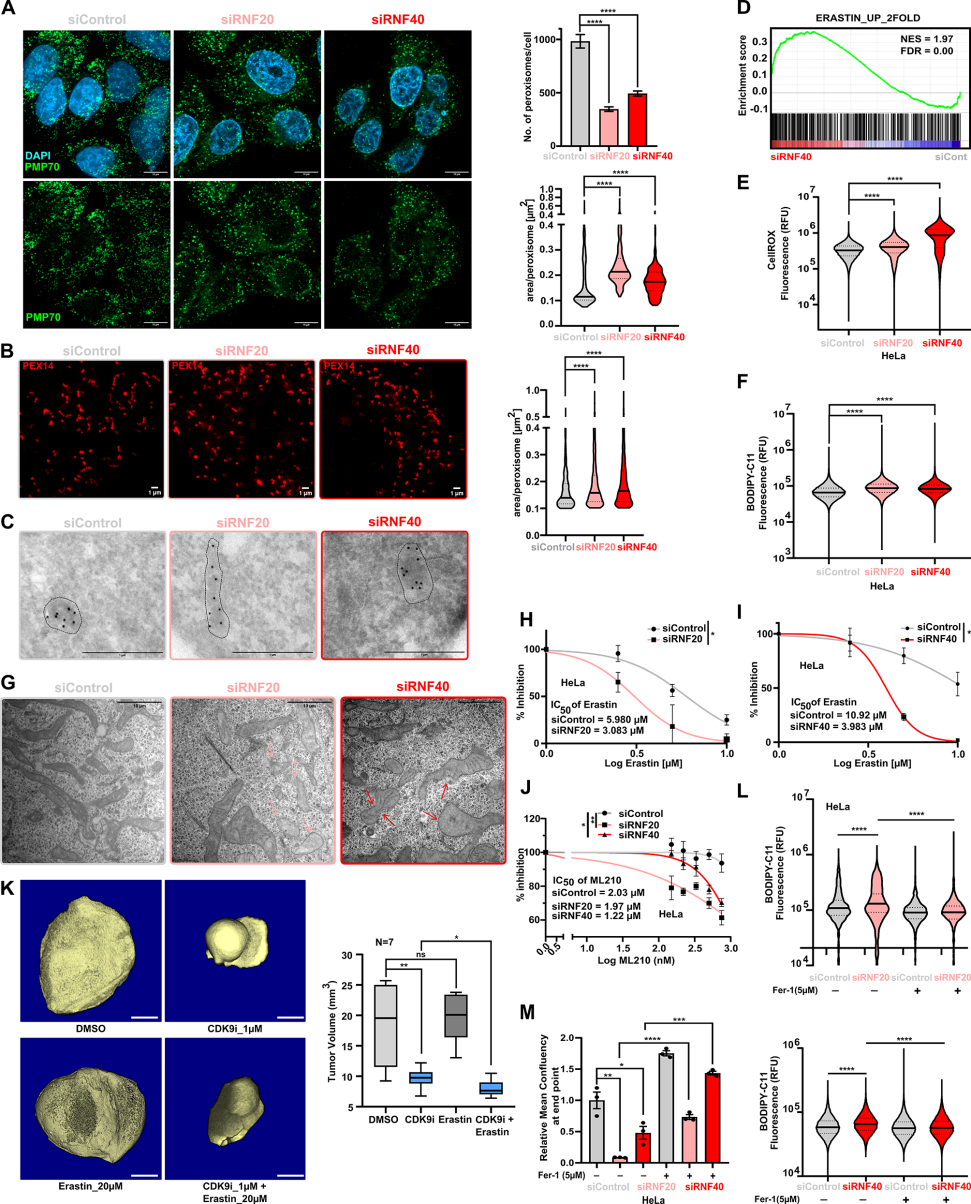
Dysregulation of peroxisome genes upon H2Bub1 loss leads to ferroptosis. **A**: PMP70 IF staining (left panel) showing decreased peroxisome number (upper right panel) and increase in peroxisome size (lower right panel) in siRNF20 and siRNF40 treated HeLa cells compared to siControl. Scale bar: 10 μm. **B**: PEX14 IF staining showing an increase in peroxisome size for siControl, siRNF20 and siRNF40 treated HeLa cells using STED microscopy. Quantification (Right panel). Scale Bar: 1µM. **C**: Electron microscopy showing peroxisome morphology in siControl, siRNF20 and siRNF40 treated HeLa cells. Scale Bar: 1µM. **D**: GSEA identified “Erastin_up-regulated” gene set significantly enriched in siRNF40-treated HeLa cells. **E**: RNF20 and RNF40 KD increase the intracellular levels of ROS, as measured by CellROX staining and flow cytometry analysis. **F**: RNF20 and RNF40 KD increase ferroptosis in HeLa cells, as measured by BODIPY-C11 staining and flow cytometry analysis. **G**: Electron microscopy showing ruptured mitochondrial membrane and loss of cristae in siControl, siRNF20 and siRNF40 treated HeLa cells. **H-J**: Sensitization of HeLa cells to ferroptosis induction with increasing concentration of Erastin upon siRNF20 (**H**) or siRNF40 (**I**) treatment and with increasing concentration of ML210 upon siRNF20 and siRNF40 treatment (**J**). IC50 values were calculated for the respective KDs and control conditions. **K**: CAM assay of HeLa cells treated with DMSO, CDK9i, and Erastin for 48 h before inoculation in eggs. The micro-CT scans of the tumors (right panel) and representative quantifications of their volumes (left panel). (*N* = 7) **L-M**: Rescue of HeLa cells to ferroptosis induction with Fer-1 (5µM) upon siRNF20 and siRNF40 treatment via decreased lipid-ROS levels (**L**) and increased cell proliferation (**M**). Statistical test: **A** (upper panel): One-way ANOVA, **A** (lower panel): Kruskal-Wallis test, **E-F**,** L**: Mann-Whitney test, **H-J**: AUC followed by Student t-test, **K**,** M**: Student t-test; *p-val < 0.05, **p-val ≤ 0.01, ***p-val ≤ 0.001, ****p-val ≤ 0.0001. Error bars: Standard error of the mean (SEM). All experiments were performed in biological triplicates per condition

As interfering with the H2Bub1 signaling reduces the expression of peroxisomal genes protecting the CC cells from ferroptosis, we questioned if RNF20/RNF40 loss would similarly affect peroxisome phenotype as observed in *PEX5*, *PEX6*, and *PRDX5* KDs. We therefore performed immunofluorescence staining for PEX14 and PMP70 upon siRNF20 or siRNF40 treatment of CC cells. As expected from our previous experiments, both losses of RNF20 or RNF40 led to impaired peroxisomes, as depicted by both reduced peroxisome numbers and enlarged peroxisomes (Fig. 5A; S5A-C). Additionally, we used STED microscopy, in order have better insights into peroxisome morphology, and performed IF staining of PEX14 in siControl, siRNF20 and siRNF40 treated HeLa cells showing increase in size of peroxisomes, thereby impaired peroxisome biogenesis (Fig. 5B). In-line to the previous findings, electron microscopy indeed showed elongated peroxisomes in KD conditions as compared to control HeLa cells; which indicates impaired ability of peroxisomes to undergo fission and regulate their function [67] (Fig. 5C). To consolidate the association of H2Bub1 loss with peroxisome dysregulation and ferroptosis, we compared the mRNA-seq data of siRNF40-treated HeLa cells with publicly available datasets from Gene Expression Omnibus (GEO, GSE104462). Strikingly, RNF40 KD treatment strongly enriched gene signatures induced by erastin treatment (Fig. 5D), a prominent ferroptosis inducer [68]. In line with this, increased levels of ROS (Fig. 5E, S5D) as well as lipid peroxidation levels were detected in HeLa (Fig. 5F, S5E) and SiHa (Fig. S5F) cells upon siRNF20 and siRNF40 treatment. To validate the functional involvement of H2Bub1 signaling in ferroptosis, we treated CC cells with a CDK9 inhibitor (CDK9i, BAY-1251152) or a proteasome inhibitor (Bortezomib), two inhibitors known to impair the ubiquitination on H2B efficiently. Both treatments with CDK9i or Bortezomib increased cellular lipid peroxidation levels in HeLa cells (Fig. S5G), further supporting the causal relationship between loss of H2Bub1, lipid peroxidation, and ferroptosis. Additionally, we observed increased loss of cristae and ruptured mitochondrial membrane, a prominent characteristic of ferroptosis, in siRNF20-, siRNF40- compared to siControl-treated HeLa cells [65] by electron microscopy (Fig. 5G). As the sole interference with H2Bub1 signaling was enough to induce ferroptotic phenotypes in CC cells, we reasoned that loss of RNF20 and RNF40 should sensitize the cells to ferroptosis by Erastin and ML210 treatment. Indeed, siRNF20 and siRNF40 pronouncedly reduced the resistance of CC cells to Erastin and ML210-induced cell death (Fig. 5H, I and J, S5H). Furthermore, H2Bub1 loss upon CDK9i along with sensitization to Erastin treatment in HeLa cells showed reduced tumor growth in the chorioallantoic membrane (CAM) assay (Fig. 5K). On the contrary, Ferrostatin-1 (Fer-1), a quencher of free iron, reverted the cell’s ferroptotic phenotypes upon loss of RNF20 and RNF40 by decreasing lipid peroxidation levels (Fig. 5L) and increasing cell confluency (Fig. 5M, S5I). Taken together, our investigations revealed a previously unknown mechanism of RNF20 and RNF40 supporting the peroxisomal transcriptional program, thereby contributing to oxidative stress homeostasis and ferroptosis suppression in CC cells.

## Discussion

CC is one of the most common female cancers across the globe. The development of novel targeted drug therapies and personalized treatments to treat CC is urgently needed. Through its E3 ligase activity, the RNF20/RNF40 heterodimeric complex catalyzes H2Bub1 deposition and epigenetically regulates gene expression via chromatin remodeling [33], transcription elongation [69], DNA repair [70] and DNA replication [71, 72]. Our group has previously explored the tumor-supportive role of H2Bub1 in HER2^+^-BC, TNBC, and colorectal cancer. In the present study, we report an additional previously unknown function of RNF20 and RNF40 promoting CC fitness through peroxisome-mediated ROS balancing and ferroptosis suppression.

Past observations made in hepatocellular carcinoma [41], mixed-lineage leukemia (MLL) [42], HER2-driven mammary carcinoma [43], colorectal cancer [44] and TNBC [45] demonstrated an association between high *RNF20* and *RNF40* levels and worse cancer disease outcomes. Our analyses of TCGA-based and single-cell RNA sequencing datasets extended the correlative behavior of H2Bub1 signaling activity and poor disease prognosis in CC patients. This observation is in line with an elegant study by Eckhardt et al. on the HPV protein interactome [73]. Here, the authors reported an active cooperation of viral proteins with RNF20/RNF40 to enact oncogenic functions. In parallel, they described an independent mechanism of viral-host factors interaction protecting cells against oxidative stress by stimulating NRF2 transcriptional activity, pointing to the necessity for infected tumor cells to counteract increasing ROS. Our in vitro functional assays confirmed the central function of the RNF20/RNF40 CC cell’s fitness and aggressiveness.

Performing differential gene expression analysis in HeLa cells and TCGA-CESC patients, we identified several RNF20 and RNF40-dependent gene signatures, the peroxisome transcriptional program being one consistently associated with high H2Bub1 signaling activity in the three studied systems. Peroxisomes help in scavenging ROS and support both energy production by fatty acid breakdown as well as the biosynthesis of lipids. Amongst the genes, we found a significant down-regulation of *PRDX5*, *PEX6*, and *PMVK* in KD conditions. *PRDX5* is a gene belonging to the peroxiredoxin family, whose functions rely on the maintenance of cellular ROS homeostasis [74]. In the context of cancer, *PRDX5* is often linked to poor disease outcomes. For instance, it was recently reported that *PRDX5* overexpression leads to EMT phenotype in gastric cancer [75]. In breast cancer, *PRDX5* interacts with the tumor-suppressor BRCA2 silencer, thereby increasing *BRCA2* expression under oxidative stress situations [76]. Importantly, *PRDX5* overexpression in cancer cells helps in scavenging excess ROS production and, therefore, plays a tumorigenic role [77]. Loss of *PRDX5* has a profound consequence on ROS levels and cell toxicity [78, 79] and is therefore very likely to be involved in the impaired phenotype upon *RNF20*/*RNF40* loss. Little is known about the function of the other H2Bub1-dependent peroxisome-related genes *PEX6* and *PMVK* in cancers. PEX6 forms a complex with PEX1 at the membrane of peroxisomes and is responsible for recycling the cargo transporter PEX5, and, therefore, plays a crucial role in peroxisome homeostasis. Our investigations identified a function of PEX6 and PVMK supporting CC cancer cell fitness in vitro. These data align with a former study by Dahabieh and colleagues reporting that *PEX6* loss leads to increased patient survival in lymphoma, lung cancer, and melanoma by combating conventional therapy resistance [80]. Also in endometrioid clear cell and high-grade serous epithelial ovarian carcinoma, high *PEX6* expression was associated with low progression-free survival of patients [81]. The PMVK is a member of the mevalonate pathway and is involved in the synthesis of isoprenoids (intermediate products of cholesterol and sterol biosynthesis) [82]. Interestingly, PVMK was shown to stimulate the Wnt-signaling activity by inhibiting CK1 and directly promoting β-catenin nuclear localization via S184 phosphorylation [83]. Luo et al. recently reported the repression of ferroptosis by LINC01606 under β-catenin stimulation, providing a plausible mechanism for CC phenotypic impairment upon PVMK loss [84]. In agreement, *PMVK* loss also leads to enhanced radiosensitivity in lung cancer cells [85] and apoptosis in various cancer cells [86]. Taken together, the identified H2Bub1-dependent peroxisome signature has strong relevance for the maintenance of cancer cells aggressiveness and resistance to oxidative conditions.

Peroxisomes play a critical role in lipid biosynthesis and ROS metabolism. This places the organelle at the crossroads of cellular homeostasis and programmed cell death via ferroptosis [87]. For example, two recently published studies have elegantly shown that the pathway for ether phospholipid production in peroxisomes is necessary for optimal ferroptosis induction [88, 89]. However, to date, no association between ROS-balancing peroxisomal function and ferroptosis modulation has been reported. In this study, we hypothesized that dysregulation of peroxisomal genes due to interference with H2Bub1 signaling could lead to increased ferroptosis. Typical hallmarks of ferroptosis include mitochondrial disruption, loss of cristae, increase in mitochondrial outer membrane densities, lipid peroxidation, increase in intracellular ferrous ions, and increase in ROS [90]. Interestingly, Wang and colleagues previously reported that loss of H2Bub1 by RNF20 KD results in increased ferroptosis in lung cancer cells by downregulating *SLC7A11* [91]. Specifically, the authors described a mechanism where the loss of H2Bub1 is driven by P53/USP7 leading to decreased *SLC7A11* expression. However, despite clear induction of ferroptosis in RNF20 KD cells, we could not identify any consistent regulation of *SLC7A11* upon siRNF20 or siRNF40 treatments. In contrast, both KDs led to robust dysregulation of peroxisomal genes involved in ROS management that are deeply involved in ferroptosis induction through increased ROS and lipid peroxidation. Interestingly, apart from H2Bub1, other ubiquitin-based epigenetic mechanisms are responsible for regulating ferroptosis. For instance, Zhang and colleagues reported that the deubiquitinase BAP1 specifically removes ubiquitin moieties from histone H2A, an epigenetic mark generally responsible for repressing gene expression, specifically at the promoter region of *SLC7A11*. As a consequence, BAP1 represses *SCL7A11* expression and elevates lipid peroxidation in the cells, ultimately leading to ferroptosis induction [92]. As such, this additional level of ubiquitin-mediated gene regulation might partially explain the discrepancies in *SLC7A11* regulation by H2Bub1 in the study by Wang et al. In contrast, the findings of our study show that resistance to ferroptosis in CC is driven by peroxisome-dependent functions regulated by the RNF20/RNF40/H2Bub1 axis.

## Conclusion

Taken together, high RNF20 and RNF40 levels are associated with poor patient survival and play a tumor-supportive role in CC. The RNF20/RNF40/H2Bub1-axis mediates increased peroxisome functions and consequently stimulates aggressiveness in CC through resistance to ferroptosis. Therefore, RNF20 and RNF40 may act as a new biomarker in CC, and targeting this axis might provide new treatment options along with conventional therapies.

## Electronic supplementary material

Below is the link to the electronic supplementary material.

Supplementary Material 1

Supplementary Material 2

Supplementary Material 3

Supplementary Material 4

Supplementary Material 5

Supplementary Material 6

Supplementary Material 7

## Data Availability

mRNA-seq data have been deposited at ArrayExpress (https://www.ebi.ac.uk/arrayexpress/) (accession number: E-MTAB-13917).
