# Supplementary material for: RNF20/RNF40 supports the aggressive behavior in cervical cancer by regulating a peroxisome-based anti-ferroptotic mechanism
Source: Cell Commun Signal. 2025 Jul 1;23:304. doi: 10.1186/s12964-025-02279-9 (PMC12210586; doi:10.1186/s12964-025-02279-9)
Supplement: Supplementary file 1 — Supplementary Material 1 [file 12964_2025_2279_MOESM1_ESM.docx]

**Supplementary Materials**

***Functional assays***

All experiments were performed in biological triplicates. The results were plotted with GraphPad Prism v8.0.1.

*Proliferation kinetics*: 20,000 cells per well were seeded in a 24-well plate. 24 hr post-transfection, cell confluency was recorded every 24 or 48 hr over the period of 6 days using a Celigo® S imaging cytometer (Nexcelom Bioscience LLC). Treatment with Erastin (ferroptosis inducer) was performed 24 hr post-transfection for a duration of 24 hr (Erastin = 0 μΜ, 0.25 µM, 5 µM and 10 µM).

*Proliferation endpoint analysis*: 20-25 000 cells per well were seeded in a 24-well plate. 8-10 days after transfection, cells were fixed with methanol for 20 min, stained with 0.25 % crystal violet (Sigma) in 20% methanol for 20 min and washed with tap water. Treatment with Erastin (ferroptosis inducer) was performed after 24 hr post-transfection for a duration of 24 hr (Erastin=0 μΜ, 0.25 µM, 5 µM and 10 µM). Treatment with Ferrostatin-1 (ferroptosis inhibitor) was performed after 24 hr post-transfection (Ferrostatin-1=0 μΜ and 5 µM). Finally, stained cells were scanned using an EPSON perfection V850 PRO scanner.

*Clonogenic assay*: 48 h post-transfection, 500 cells were seeded in a 12-well plate. 10-12 days after seeding, colonies were washed with PBS, fixed with methanol for 20 min, stained with 0.25 % crystal violet in 20% methanol for 20 min and washed with tap water. Finally, stained colonies were scanned using an EPSON perfection V850 PRO scanner. The number and size of colonies were assessed using ImageJ.

*Tumorsphere formation assay*: At 48 hr post-transfection, 1,000 cells per well were seeded in a low adherent 96-well plate. 15 days after seeding, spheres were photographed with a Celigo® S imaging cytometer (Nexcelom Bioscience LLC). The number and size of spheres were assessed using ImageJ.

**Protein isolation and western blot analyses**

For protein isolation, 80,000 cells were seeded per well in a 12-well plate and reverse transfected with siRNA. After 72 hr, cells were washed once with PBS and 100 μl of RIPA buffer was added to each well (12-well plate). RIPA buffer = Radioimmunoprecipitation Assay Buffer: 10 mM Tris-Cl pH 8, 1 mM EDTA, 1% v/v Triton X-100, 0.1% sodium deoxycholate, 0.1% SDS, 140 mM NaCl, supplemented with protease and phosphatase inhibitors (1 μM activated orthovanadate, 10 mM β-glycerophosphate disodium salt hydrate, 10 mM Pefablock, 10 mM N-Ethylmaleimide, 1 mM Aprotinin/Leupeptinin, 1 mM NaF, 1 μM iodoacetic acid). After 10 min incubation on ice, cells were scraped and lysates were sonicated for ten cycles (each 30s ON/ 30s OFF) using a Bioruptor (Diagenode). Protein concentration of the cell lysates was assessed with the BCA method (Pierce). 6x Laemmli buffer (375 mM Tris/HCl, 10% SDS, 30% glycerol, 0.02% bromophenol blue, 9.3% DTT) was added to each lysate and cooked at 95°C for 5 min before protein separation with a 10 to 12% polyacrylamide gel. Proteins were transferred to a nitrocellulose membrane (0.45 µm pore, Immobilon, Millipore), blocked with 5% skimmed milk in TBS-T for 1 hour and incubated with primary antibody overnight at 4°C. The day after, the membrane was washed with TBS-T, and incubated for 1h with a secondary antibody at room temperature. After a final wash step, protein detection was achieved with the Millipore substrate in an Intas Chemostar Imager (Intas Science Imaging). Used primary antibodies are listed in Table S4-5.

***RNA isolation and real-time quantitative PCR (RT-qPCR)***

For RNA isolation, 80,000 cells per well in a 12-well plate were reverse transfected with siRNA. 72 hr after siRNA treatment, cells were washed with PBS and lysed in 500 µl EXTRAzol (EM30-100, BLIRT). Lysates were then collected and RNA was extracted, as previously described (1,2). Reverse transcription of 1 µg RNA was performed using M-MuLV reverse transcriptase (GeneON) with random primers according to the manufacturer’s instructions. The expression of specific genes was finally estimated by quantitative real-time PCR using a CFX Connect™ Real-Time System (Bio-Rad). Gene expression levels were normalized relative to the *RPLP0* housekeeping gene (all RT-qPCR experiments). RT-PCR program: 1x 2 min-95 ^o^C, 40x 10 sec-95 ^o^C followed by 1x 30 sec-60°C. Primers (Table S2) were designed using the online tool https://www.ncbi.nlm.nih.gov/tools/primer-blast/ and were ordered from Sigma-Aldrich (Germany). The results were finally plotted with GraphPad Prism v8.0.1.

**Fixation of HeLa cells for STED Microscopy**

The HeLa cells were KD with siRNA-mediated transfection protocol using DharmaFECT-1 as described previously. 72 hr after transfection the cells were washed 3X with PBS for 5 min. 0.1-0.5% Triton X-100 in PBS pH 7.4 was added for 10 min. Wash 3X with PBS for 5 min. The samples were incubated in PBT (1-3% BSA + 0.1% Tween 20 in PBS pH 7.4) for 1 hr. The samples were incubated with PEX14 primary antibody (1:200; diluted in PBT) overnight in a humid chamber. Wash 3X with PBS for 5 min, followed by 0.1% Triton X-100 for 5 min. The samples were incubated in PBT for 30 min, followed by secondary antibody (1:200; diluted in PBT) and DAPI (1:1000; diluted in PBT) as a counter stain for 1 hr in a humid chamber. Wash 3X with PBS for 5 min. The coverslips were mounted in 7 µl Mowiol mounting medium and STED microscopy was performed.

**CAM Assay tumor paraffin embedding**

Excised tumors were briefly rinsed five times in water and then transferred to 35% and 70% ethanol (1 hour each). For staining and fixation, tumors were placed overnight at room temperature (RT) under slow rotation in a 4% paraformaldehyde solution (PFA, Serva Electrophoresis) in phosphate-buffered saline, pH 7.4, containing 0.7% phosphotungstic acid solution (PTA, Sigma-Aldrich Corp.) diluted in 70% ethanol. Samples were then briefly rinsed in water and stored in fresh 70% ethanol. For further μCT analysis, the PTA-stained tumors were dehydrated with ascending ethanol series and embedded in paraffin. Further, micro-CT scans were performed.

**Electron Microscopy via Immunolabeling using Protein-A gold**

**
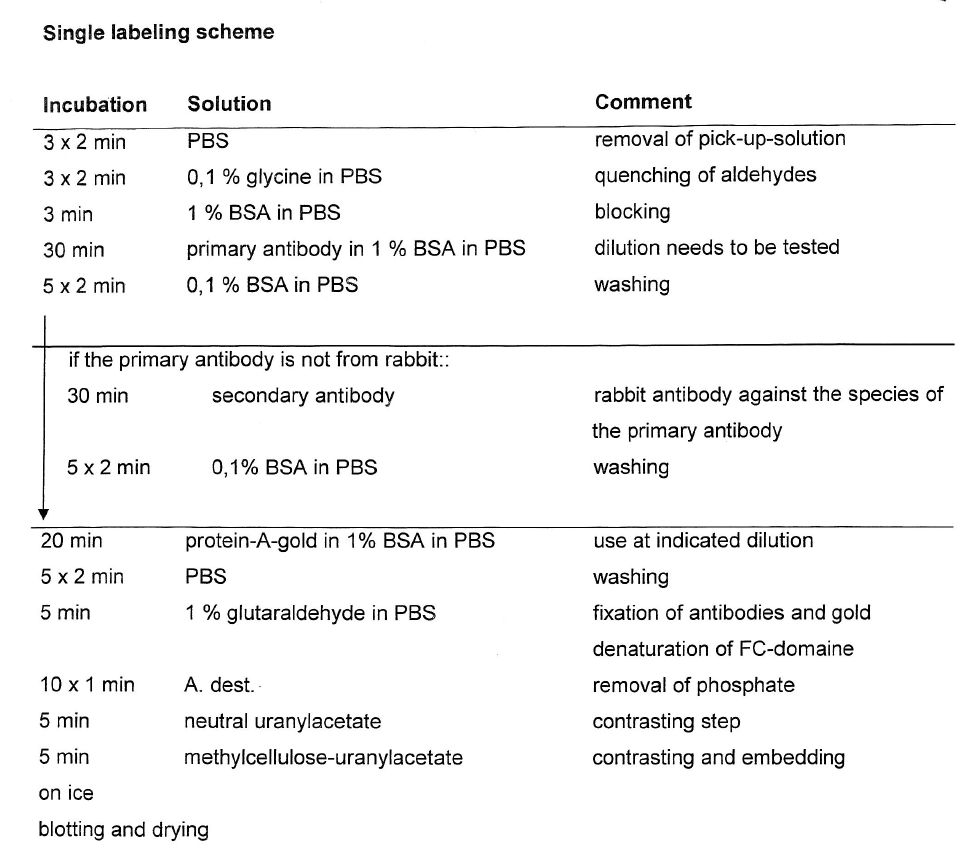
**

***Supplementary Tables***

**Table S1: siRNAs used in this study**.

| **Gene** | **siRNA** | **Cat. Number** |
| --- | --- | --- |
| Non-Τargeting #5 | UGGUUUACAUGUCGACUAA | D-001210-05-50  Dharmacon |
| *RNF40* | #1 GAGAUGCGCCACCUGAUUA | D-006913-01  Dharmacon |
|  | #2 GAUGCCAACUUUAAGCUAA | D-006913-02  Dharmacon |
|  | #3 GAUCAAGGCCAACCAGAUU | D-006913-03  Dharmacon |
|  | #4 CAACGAGUCUCUGCAAGUG | D-006913-04  Dharmacon |
| *RNF20* | #1 CCAAUGAAAUCAAGUCUAA | D-007027-01  Dharmacon |
|  | #2 UAAGGAAACUCCAGAAUAU | D-007027-02  Dharmacon |
|  | #3 GCAAAUGUCCCAAGUGUAA | D-007027-03  Dharmacon |
|  | #4 AGAAGAAGCUACAUGAUUU | D-007027-04  Dharmacon |
| *PEX5* | #1 GCACACGGCCAGUGACUUU | D-015788-01 Dharmacon |
|  | #2 CGUCAGCUACCUAUGAUAA | D-015788-02 Dharmacon |
|  | #3 CUAUAGAGUUGCAGGCAGA | D-015788-03 Dharmacon |
|  | #4 GCGGAGGUGUCUGGAGCUA | D-015788-04 Dharmacon |
| *PEX6* | #1 GGCCAGAGCUCAUUAACAU | D-004902-01 Dharmacon |
|  | #2 CAAAGGAAGCUGCUCAUUG | D-004902-02 Dharmacon |
|  | #3 AGAGUUACACAUCGAAAUU | D-004902-19 Dharmacon |
|  | #4 UGGAAAUUAUGACGGUGUU | D-004902-20 Dharmacon |
| *PRDX5* | #1 GGAGACAGACUUAUUACUA | D-019102-01 Dharmacon |
|  | #2 GGGAAUCGACGUCUCAAGA | D-019102-02 Dharmacon |
|  | #3 GGUACAGGAUGGCAUAGUG | D-019102-03  Dharmacon |
|  | #4 GCUCAGCGGGCUAUAUACU | D-019102-04 Dharmacon |

**Table S2: RT-PCR and ChIP-qPCR primers for gene expression analyses used in this study**.

| **Gene name** | **Forward (5‘-3‘)** | **Reverse (5‘-3‘)** | **Species** | **Reference** |
| --- | --- | --- | --- | --- |
| ***RPLP0*** | GATTGGCTACCCAACTGTTG | CAGGGGCAGCAGCCACAAA | Human | (3) |
| ***RNF20*** | TGGCCAAGCAGGAAGAAG | ACGCTCTGACATGAGCTTGA | Human | This study |
| ***RNF40*** | AGTACAAGGCGCGGTTGA | GAAGCAGAAAACGTGGAAGC | Human | (3) |
| ***PEX5*** | TGGGAGTCCTTTTCAACCTG | CGCCTAGCTTATTCCACAGC | Human | This study |
| ***PEX6*** | CTGTGACCCCCTGGAAATGG | ACTCTCTGGCAAATGGAGGC | Human | This study |
| ***PRDX5*** | GCGGGCTATATACTCGTCGG | AGGAGCCGAACCTTGATTGG | Human | This study |
| ***PMVK*** | CTGTTCAGCGGCAAGAGGA | GGGAGATGCCCTCCACAATC | Human | This study |
| ***PEX6_H2Bub1_ChIP*** | GGGATGATATGGGGCACGAG | AAAGGACTGGGGTCAGGAGT | Human | This study |
| ***PRDX5_H2Bub1_ChIP*** | CCTCTTTCCGGCAGGTTCTC | GGGAGGGGTGGAGGAAGTAA | Human | This study |
| ***PMVK_H2Bub1_ChIP*** | GTCAGGGTTTGGAGGTGAGG | CTCTTGAAGGCCAGTGTGGT | Human | This study |

**Table S3: cell lines used in this study**.

| **Cell line** | **HeLa (CCl-2 ^TM^)** | **SiHa (HTB-35 ^TM^)** |
| --- | --- | --- |
| **Tissue of origin** | 31 years old, black female with Adenocarcinoma | 55 years old, female, Squamous cell carcinoma |
| **Morphology and growth properties** | adherent, epithelial cells | epithelial, adherent |
| **Supplier** | ATCC | ATCC |
| **Recommended medium** | MEM (Biowest) | MEM (Biowest) |
| **HPV status** | HPV-18 sequences present | HPV-16 sequences present |
| **pRB status** | +ve | +ve |
| **p53 status** | Low expression | +ve |

**Table S4: List of primary antibodies**.

|  | **WB (dilutions)** | **IF**  **(dilutions)** | **cat. number, company** |
| --- | --- | --- | --- |
| **GAPDH** | (1:1000) |  | #607901, Biolegend |
| **H2Bub1** | (1:1000) |  | #5546, CST |
| **PEX14** |  | 1:500 for IF; 1:200 for STED | 10594-1-AP, proteintech |
| **PMP70** |  | 1:1000 | AF488, Abcam |
| **H2B** | (1:1000) |  | 12364S, CST |
| **PEX6** | (1:1000) |  | A10459, Abclonal |
| **PRDX5** | (1:1000) |  | A1269, Abclonal |
| **PMVK** | (1:3000) |  | A13865, Abclonal |
| **Catalase** | (1:1000) |  | 200-401-051, Rockland |

**Table S5: List of secondary antibodies.**

|  | **WB (Dilution)** | **IHC (Dilution)** | **Cat.number, company** |
| --- | --- | --- | --- |
| **HRP-anti-rabbit IgG** | (1:10 000) |  | 211-032-171,  Dianova |
| **Anti-rat IgG** | (1:20 000) |  | Dianova |
| **Abberior STAR RED goat anti-rabbit IgG** | (1:200) |  | STRED-1002-100UG, Abberior |

**Table S6: List of pathways enriched in high *RNF20* and *RNF40* expressing CC cells.**

| **Pathways enriched in high *RNF20* expressing CC cells via gene-set enrichment analysis** | **NES** | **FDR** |
| --- | --- | --- |
| HALLMARK_EPITHELIAL_MESENCHYMAL_TRANSITION | 2.108991 | 0 |
| HALLMARK_APICAL_SURFACE | 1.977388 | 5.38E-04 |
| HALLMARK_ANDROGEN_RESPONSE | 1.863856 | 0.001185 |
| HALLMARK_MYOGENESIS | 1.822848 | 0.002635 |
| HALLMARK_ESTROGEN_RESPONSE_LATE | 1.806744 | 0.002108 |
| HALLMARK_ANGIOGENESIS | 1.666678 | 0.009984 |
| HALLMARK_COAGULATION | 1.596065 | 0.023501 |
| HALLMARK_ESTROGEN_RESPONSE_EARLY | 1.589033 | 0.022275 |
| HALLMARK_UV_RESPONSE_DN | 1.573609 | 0.024355 |
| HALLMARK_BILE_ACID_METABOLISM | 1.563268 | 0.024174 |
| HALLMARK_GLYCOLYSIS | 1.515468 | 0.037154 |
| HALLMARK_HYPOXIA | 1.454371 | 0.067242 |
| HALLMARK_CHOLESTEROL_HOMEOSTASIS | 1.434775 | 0.069283 |
| HALLMARK_IL2_STAT5_SIGNALING | 1.403645 | 0.085015 |
| HALLMARK_KRAS_SIGNALING_UP | 1.352817 | 0.125268 |
| HALLMARK_INFLAMMATORY_RESPONSE | 1.310086 | 0.170228 |
| HALLMARK_HEDGEHOG_SIGNALING | 1.308933 | 0.162085 |
| HALLMARK_G2M_CHECKPOINT | 1.296441 | 0.168313 |
| HALLMARK_XENOBIOTIC_METABOLISM | 1.282752 | 0.176447 |
| HALLMARK_APICAL_JUNCTION | 1.281992 | 0.169203 |
| HALLMARK_MTORC1_SIGNALING | 1.271195 | 0.177306 |
| HALLMARK_FATTY_ACID_METABOLISM | 1.261365 | 0.183054 |
| HALLMARK_COMPLEMENT | 1.260318 | 0.177097 |
| HALLMARK_E2F_TARGETS | 1.248119 | 0.186198 |
| HALLMARK_TNFA_SIGNALING_VIA_NFKB | 1.236864 | 0.19452 |
| HALLMARK_KRAS_SIGNALING_DN | 1.22418 | 0.204885 |
| HALLMARK_MITOTIC_SPINDLE | 1.214066 | 0.211218 |

| **Pathways enriched in high *RNF40* expressing CC cells via gene-set enrichment analysis** | **NES** | **FDR** |
| --- | --- | --- |
| HALLMARK_P53_PATHWAY | 2.072538 | 0.00 |
| HALLMARK_XENOBIOTIC_METABOLISM | 1.9687772 | 0.00 |
| HALLMARK_HYPOXIA | 1.8135651 | 0.00 |
| HALLMARK_MYOGENESIS | 1.815996 | 0.01 |
| HALLMARK_EPITHELIAL_MESENCHYMAL_TRANSITION | 1.6994385 | 0.01 |
| HALLMARK_INFLAMMATORY_RESPONSE | 1.6258023 | 0.02 |
| HALLMARK_GLYCOLYSIS | 1.5696464 | 0.04 |
| HALLMARK_APOPTOSIS | 1.509022 | 0.06 |
| HALLMARK_APICAL_SURFACE | 1.4926084 | 0.06 |
| HALLMARK_DNA_REPAIR | 1.4815618 | 0.06 |
| HALLMARK_OXIDATIVE_PHOSPHORYLATION | 1.464576 | 0.06 |
| HALLMARK_TGF_BETA_SIGNALING | 1.4520552 | 0.06 |
| HALLMARK_TNFA_SIGNALING_VIA_NFKB | 1.4307126 | 0.07 |
| HALLMARK_ESTROGEN_RESPONSE_LATE | 1.4042242 | 0.08 |
| HALLMARK_WNT_BETA_CATENIN_SIGNALING | 1.3934822 | 0.08 |
| HALLMARK_KRAS_SIGNALING_DN | 1.2798165 | 0.17 |
| HALLMARK_NOTCH_SIGNALING | 1.2807804 | 0.18 |

**CAM Assay:** The eggs for the CAM assay was purchased from VALO BioMedia.

**Supplementary References**

1. Prenzel T, Begus-Nahrmann Y, Kramer F, Hennion M, Hsu C, Gorsler T, et al. Estrogen-dependent gene transcription in human breast cancer cells relies upon proteasome-dependent monoubiquitination of histone H2B. Cancer Res. 2011;71(17):5739–53.

2. Mishra VK, Wegwitz F, Kosinsky RL, Sen M, Baumgartner R, Wulff T, et al. Histone deacetylase class-I inhibition promotes epithelial gene expression in pancreatic cancer cells in a BRD4-and MYC-dependent manner. Nucleic Acids Res. 2017;45(11):6334–49.

3. Wegwitz F, Prokakis E, Pejkovska A, Kosinsky RL, Glatzel M, Pantel K, et al. The histone H2B ubiquitin ligase RNF40 is required for HER2-driven mammary tumorigenesis. Cell Death Dis. 2020 Oct 1;11(10):873.

**Supplementary figures**

**Figure S1: Cervical cancer patient’s survival analysis. A**: Progression-free interval (PFI) analysis of H2bub1^high^ score and H2bub1^low^ score in squamous carcinoma CC patients. **B**: PFI analysis of *RNF20*^high^ and *RNF20*^low^ in squamous carcinoma CC patients. **C**: PFI analysis of *RNF40*^high^ and *RNF40*^low^ in squamous carcinoma CC patients. **D and E**: *RNF20* (**D**) and *RNF40* (**E**) expression based on the clinical stages in CC patients. **F and G**: *RNF20* (**F**) and *RNF40* (**G**) expression based on the Pathologic T stage in CC patients. **H and I**: *RNF20* (**H**) and *RNF40* (**I**) expression based on the Pathologic N stage in CC patients. **J and K**: *RNF20* (**J**) and *RNF40* (**K**) expression based on the Pathologic M stage in CC patients. (Source: TCGA-CESC, retrieved from: <https://xenabrowser.net/>). **L**: Publicly available scRNA sequencing dataset of SCC (GSE197461) reanalyzed and represented as UMAP plot. The dashed lined area identifies tumor cell clusters based on the results of a SingleR analysis. **M**: UMAP plot for tumor cells clusters expressing *KRT14* and *CDKN2A* in SCC. **N**: Publicly available scRNA sequencing dataset of ADC (GSE197461) reanalyzed and represented as UMAP plot. The dashed lined area identifies tumor cell clusters based on the results of a SingleR analysis. **O**: UMAP plot for tumor cells clusters expressing *EPCAM* and *KRT18* in ADC. Statistical test: **A-C**: Log-rank test, **D-K**: Mann-Whitney test. **p*-val<0.05, ***p*-val≤0.01, *p*-val>0.05 = not significant.

**Figure S2: *RNF20* and *RNF40* plays a tumorigenic role *in vitro*: A**: Real-time quantitative PCR (RT-qPCR) (Left panel) and Western blots along with quantification (Right panel) of *RNF20* and *RNF40* in siControl, siRNF20 and siRNF40-treated SiHa cells at 72 hr of silencing. **B**: Western Blot of H2Bub1 in siControl, siRNF20, and siRNF40-treated SiHa cells at 72 hr of silencing. Quantification is shown as a bar graph (left panel). **C–H**: Proliferation kinetics (**C, D**), colony (**E**), tumorsphere formation assay (**F**), Gap closure assay (**G**) and Boyden chamber assay (End point at 48 hr post-seeding in inserts) (**H**) in siControl, siRNF20 and siRNF40 treated SiHa cells. **I**: Western blot of H2Bub1 in CDK9 treated (CDK9i) (0.75µM) SiHa cells 48 hr after treatment (Top panel). Quantification as bar graph (Bottom panel). **J-K**: Relative Mean confluency at end-point (**J**) and colony formation assay (**K**) in CDK9i (0.75µM) treated SiHa cells. Statistical test: **A, B, D, E, F, H, I, J, K**: Student *t*-test. **C, G**: AUC followed by Student t-test; **p*-val<0.05, ***p*-val≤0.01, ****p*-val≤0.001, *****p*-val≤0.0001. Error bars: Standard error of the mean (SEM). All experiments were performed in biological triplicates per condition.

**Figure S3: Single siRNA-mediated KD of RNF20 and RNF40 showing reduced tumorigenic potential. A-B**: RT-qPCR of smart pool- and single siRNA-mediated KD showing expression of *RNF20* (**A**) and *RNF40* (**B**) in siControl, siRNF20 and siRNF40 treated HeLa and SiHa cells. **C-D**: Cell proliferation assay of smart pool- and single siRNA-mediated KD of *RNF20* (**C**) and *RNF40* (**D**) in siControl, siRNF20 and siRNF40 treated HeLa and SiHa cells. **E-F**: RT-qPCR of smart pool- and single siRNA-mediated KD showing expression of *PEX6, PRDX5* and *PMVK* in siControl, siRNF20 and siRNF40 treated HeLa (**E**) and SiHa (**F**) cells. Statistical test: **A-F:** Student *t*-test; **p*-val<0.05, ***p*-val≤0.01, ****p*-val≤0.001, *****p*-val≤0.0001. Error bars: Standard error of the mean (SEM). All experiments were performed in biological triplicates per condition.

**Figure S4: Loss of *PEX5*, *PEX6* and *PRDX5* results in impaired peroxisomes. A**: RT-qPCR of peroxisome genes in DMSO and CDK9i (0.75µM) treated SiHa cells (24 hr of treatment). **B- D**: Confocal microscopy: PMP70 IF staining (**B**) showing decreased peroxisome number (**C**) and increase in peroxisome size (**D**) in siPEX5 and siPRDX5 treated HeLa cells compared to siControl. **E**: Histograms of ROS levels in siControl, siPEX5, siPEX6 and siPRDX5-treated HeLa cells by CellRox staining. **F**: Histograms of lipid peroxidation levels in siControl, siPEX5, siPEX6 and siPRDX5-treated HeLa cells by BODIPY-C11 staining. **G**: Violin plot (left panel) and histograms (right panel) lipid peroxidation levels in siControl, siPEX5, siPEX6 and siPRDX5-treated SiHa cells by BODIPY-C11 staining. Statistical test: **A:** Student t-test, **C**: One-way ANOVA, **D**: Kruskal-Wallis test, **G**: Mann-Whitney test; **p*-val<0.05, ****p*-val≤0.001, *****p*-val≤0.0001. Error bars: Standard error of the mean (SEM). Scale Bar: 10µm. All experiments were performed in biological triplicates per condition.

**Figure S5: Loss of H2Bub1 results in impaired peroxisome function thereby inducing ferroptosis. A to C**: PEX14 IF staining (**A**) showing decreased peroxisome number (**B**) and increase in peroxisome size (**C**) for siRNF20- and siRNF40-treated HeLa cells compared to siControl. **D**: Histograms of ROS levels in siControl-, siRNF20-, siRNF40-treated HeLa cells by CellRox staining. **E**: Histograms of lipid peroxidation levels in siControl-, siRNF20-, siRNF40-treated HeLa cells by BODIPY-C11 staining. **F**: Violin plot (left panel) and histograms (right panel) of lipid peroxidation levels in siControl, siRNF20 and siRNF40 treated SiHa cells by BODIPY-C11 staining. **G**: Impairment of the H2Bub1-signaling by 24 hr treatment with a CDK9 inhibitor (BAY-1251152, 250 nM) or proteasome inhibitor (Bortezomib, 20 nM) induces ferroptosis in HeLa cells, as measured by BODIPY-C11 staining and flow cytometry analysis. **H**: Sensitization of SiHa cells to ferroptosis induction with increasing concentration of Erastin upon siRNF20 and siRNF40 treatment. **I**: Rescue of SiHa cells to ferroptosis induction with Fer-1 (5µM) upon siRNF20 and siRNF40 treatment via increased cell proliferation. Statistical test: **B**: One-way ANOVA, **C**: Kruskal-Wallis test, **F**: Mann-Whitney test. **H**: AUC followed by Student t-test, **I**: Student t-test; Error bars: Standard error of the mean (SEM). Scale Bar: 10µm. All experiments were performed in biological triplicates per condition; **p*-val<0.05, ***p*-val≤0.01, ****p*-val≤0.001, *****p*-val≤0.0001. Error bars: Standard error of the mean (SEM). All experiments were performed in biological triplicates per condition.
