# Supplementary material for: RNF20/RNF40 supports the aggressive behavior in cervical cancer by regulating a peroxisome-based anti-ferroptotic mechanism
Source: Cell Commun Signal. 2025 Jul 1;23:304. doi: 10.1186/s12964-025-02279-9 (PMC12210586; doi:10.1186/s12964-025-02279-9)

Western Blot H2Bub1

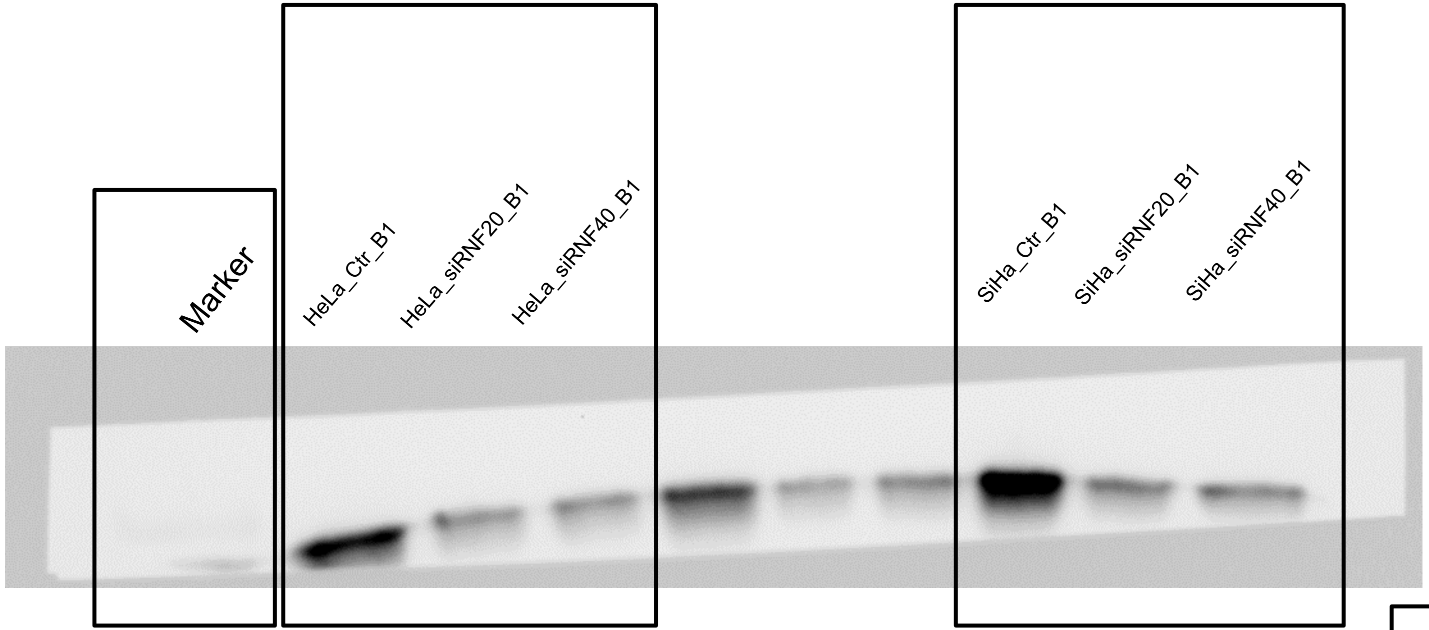

Western Blot GAPDH (loading control)

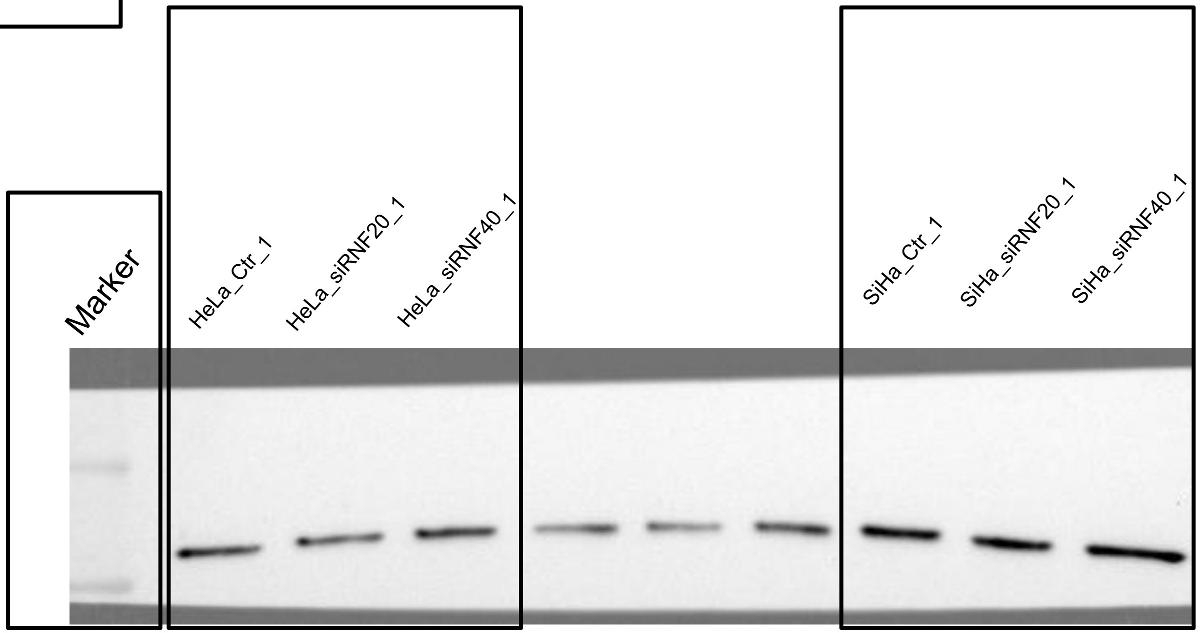

Western blot Hela RNF20 KD lysates

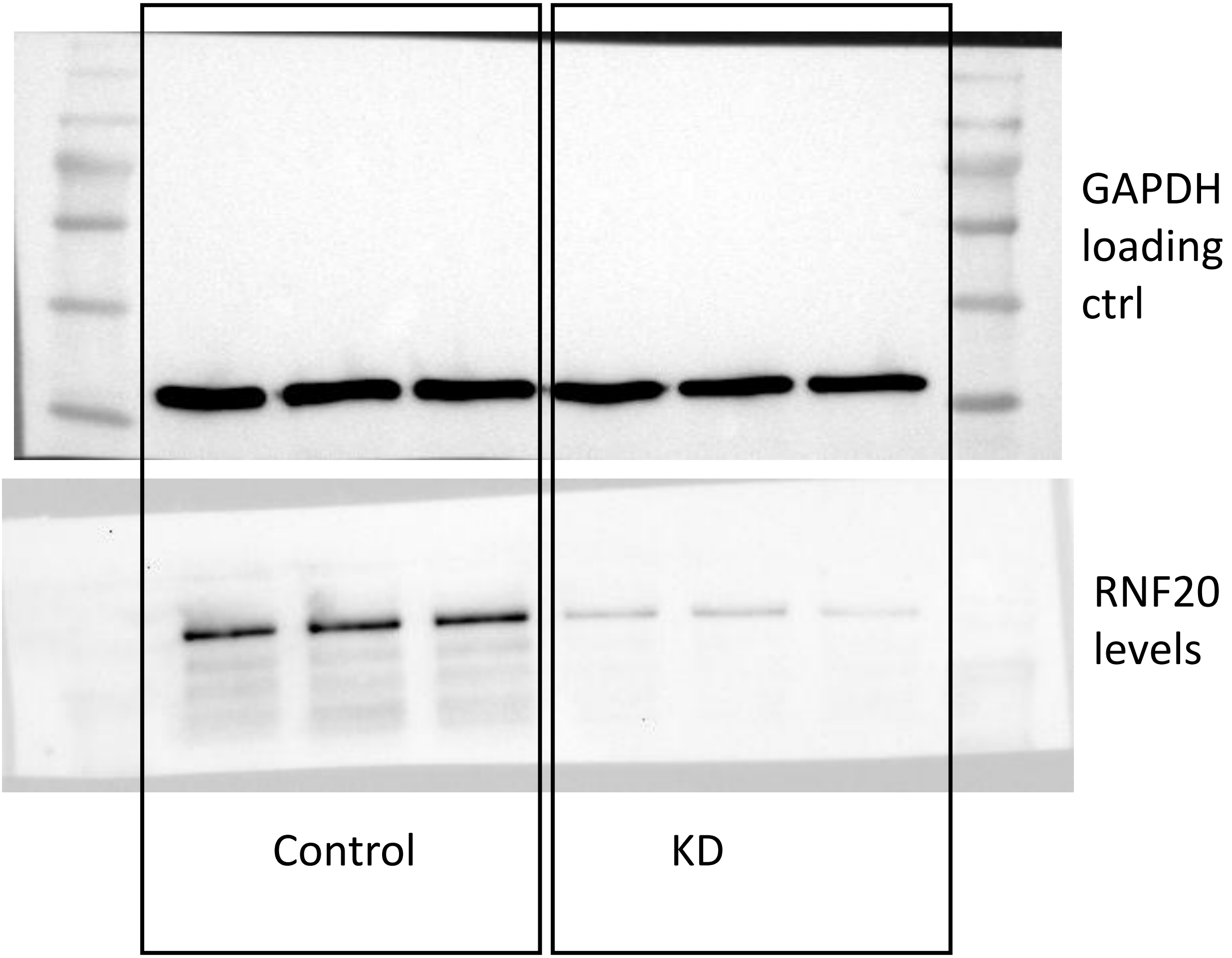

Western blot Hela RNF20 KD lysates

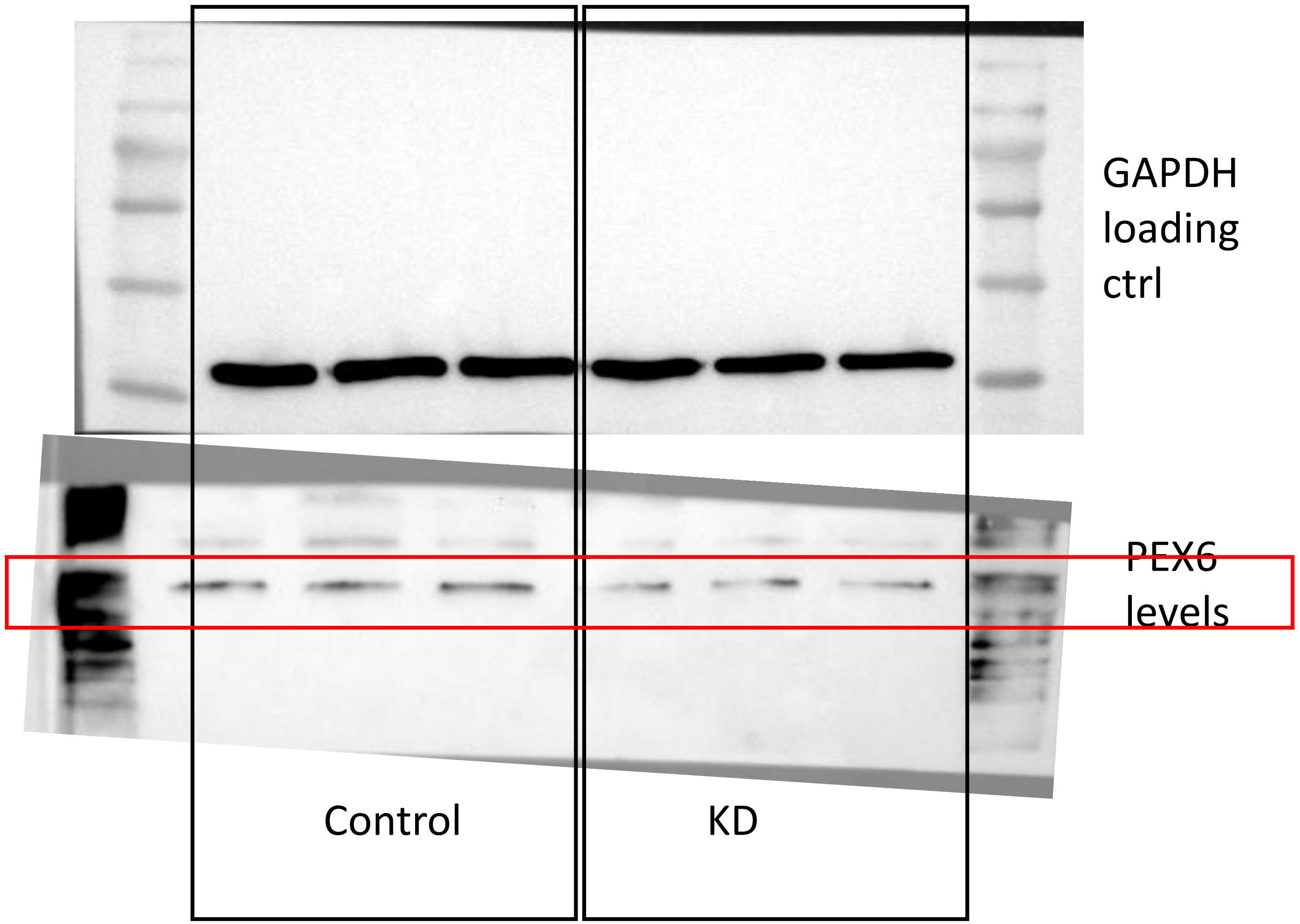

Western blot Hela RNF20 KD lysates

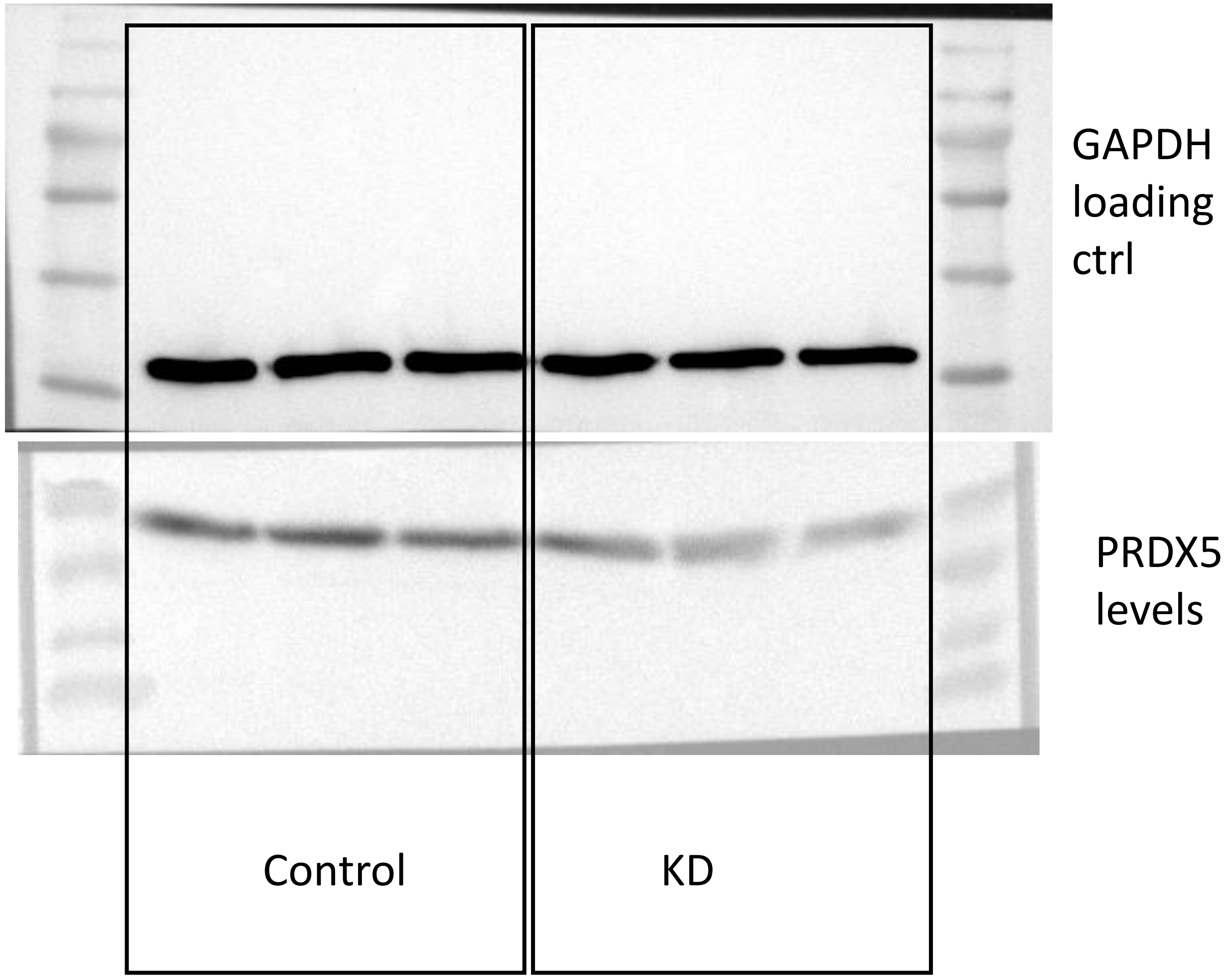

Western blot Hela RNF20 KD lysates

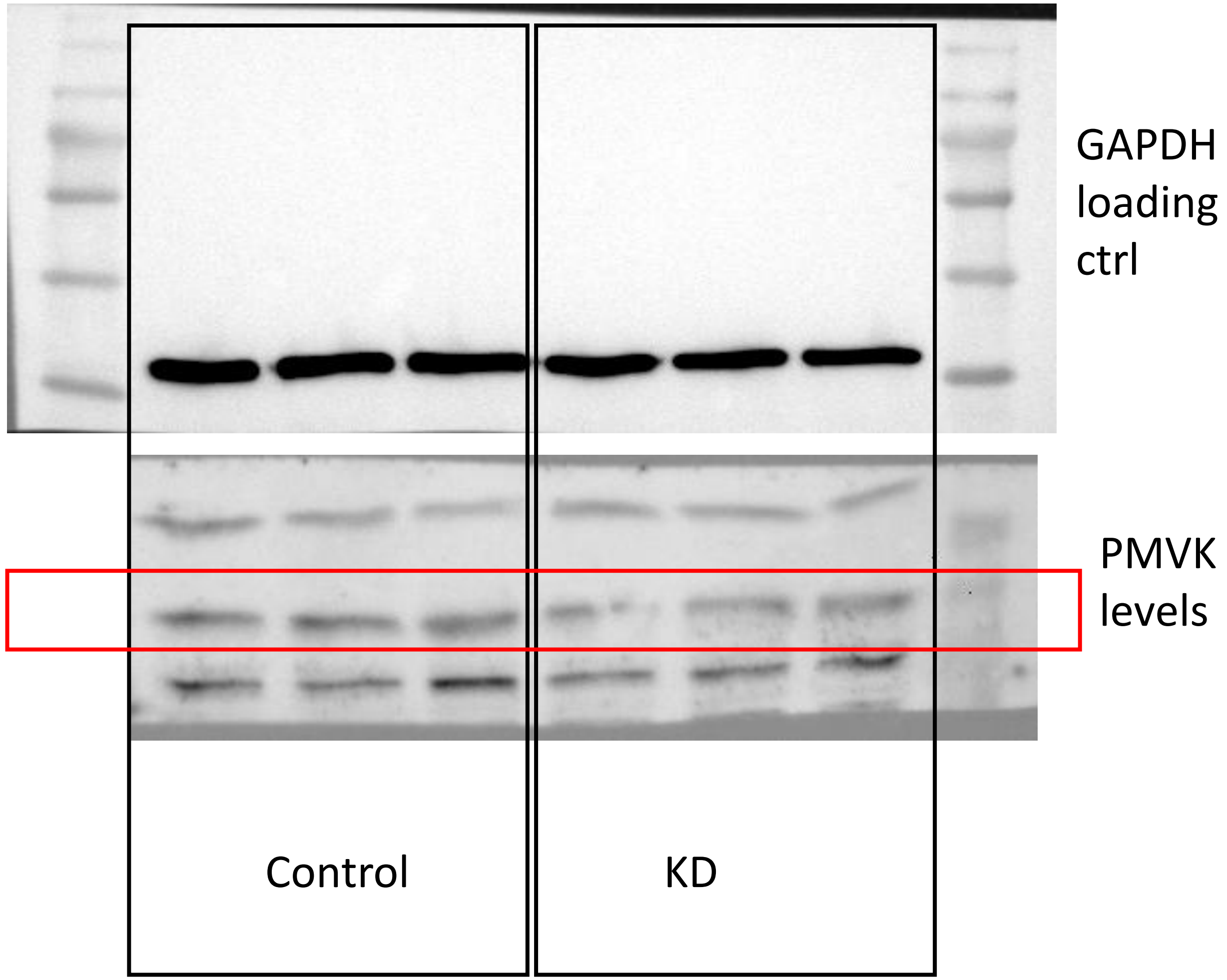

Western blot Hela RNF40 KD lysates

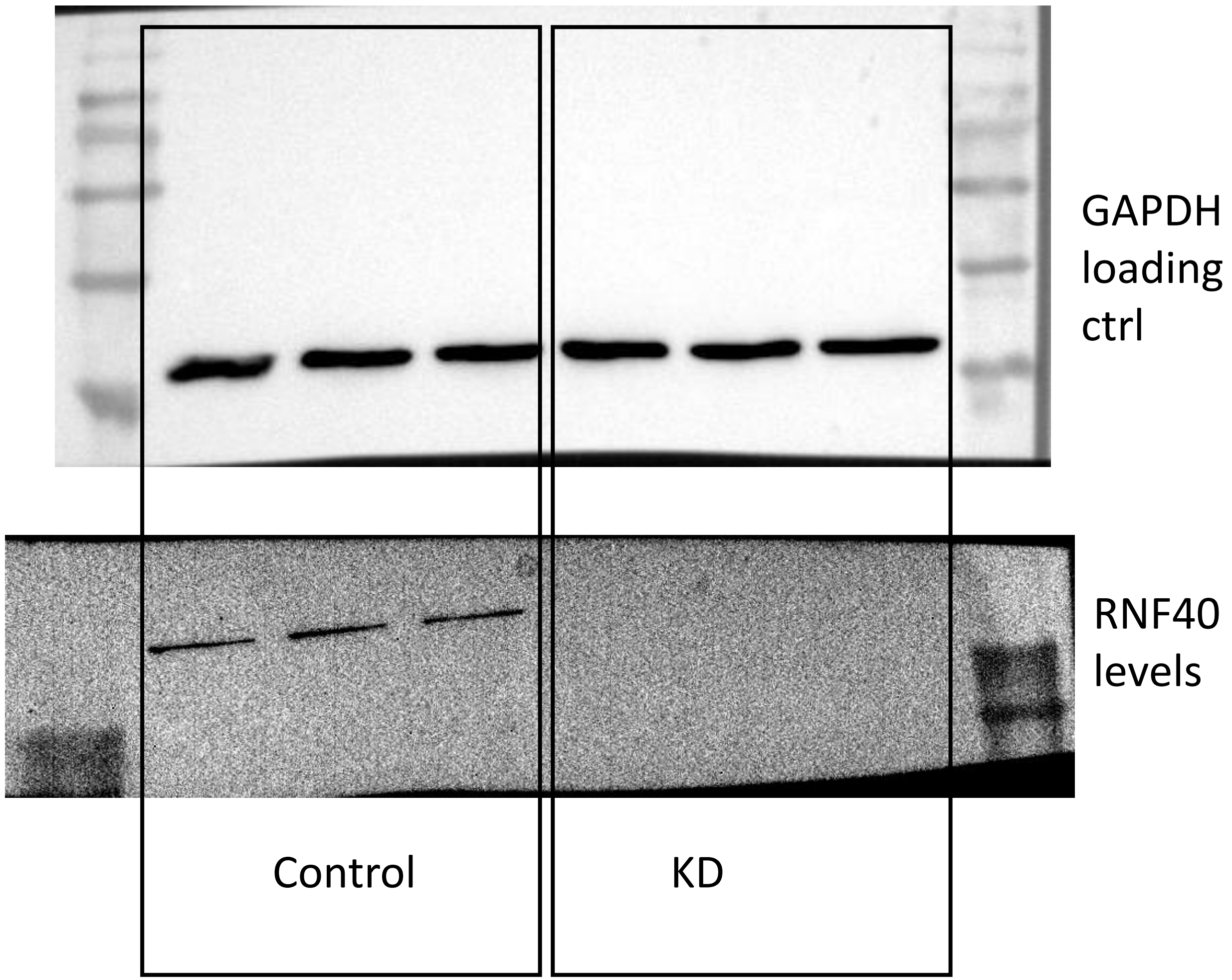

Western blot Hela RNF40 KD lysates

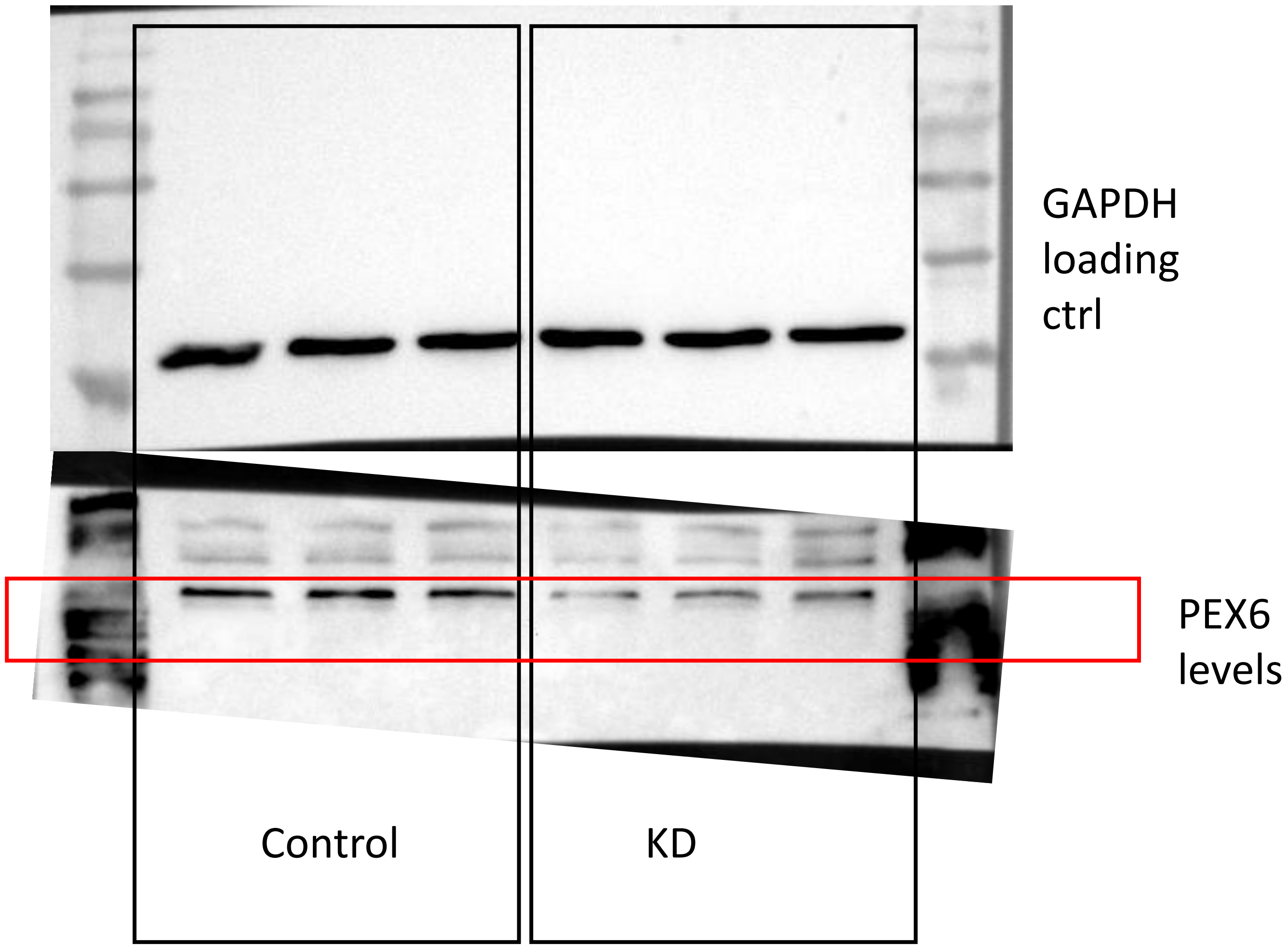

Western blot Hela RNF40 KD lysates

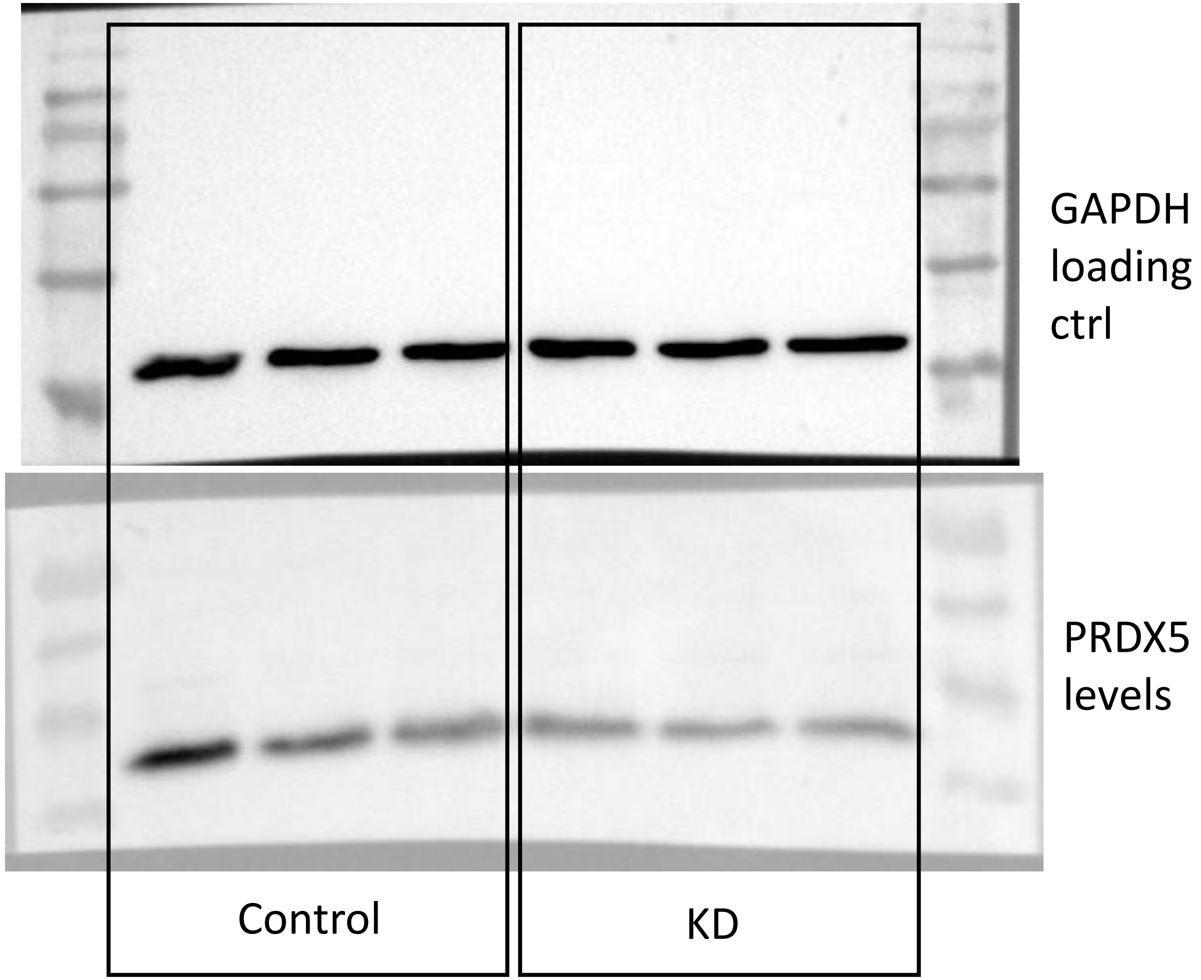

Western blot Hela RNF40 KD lysates

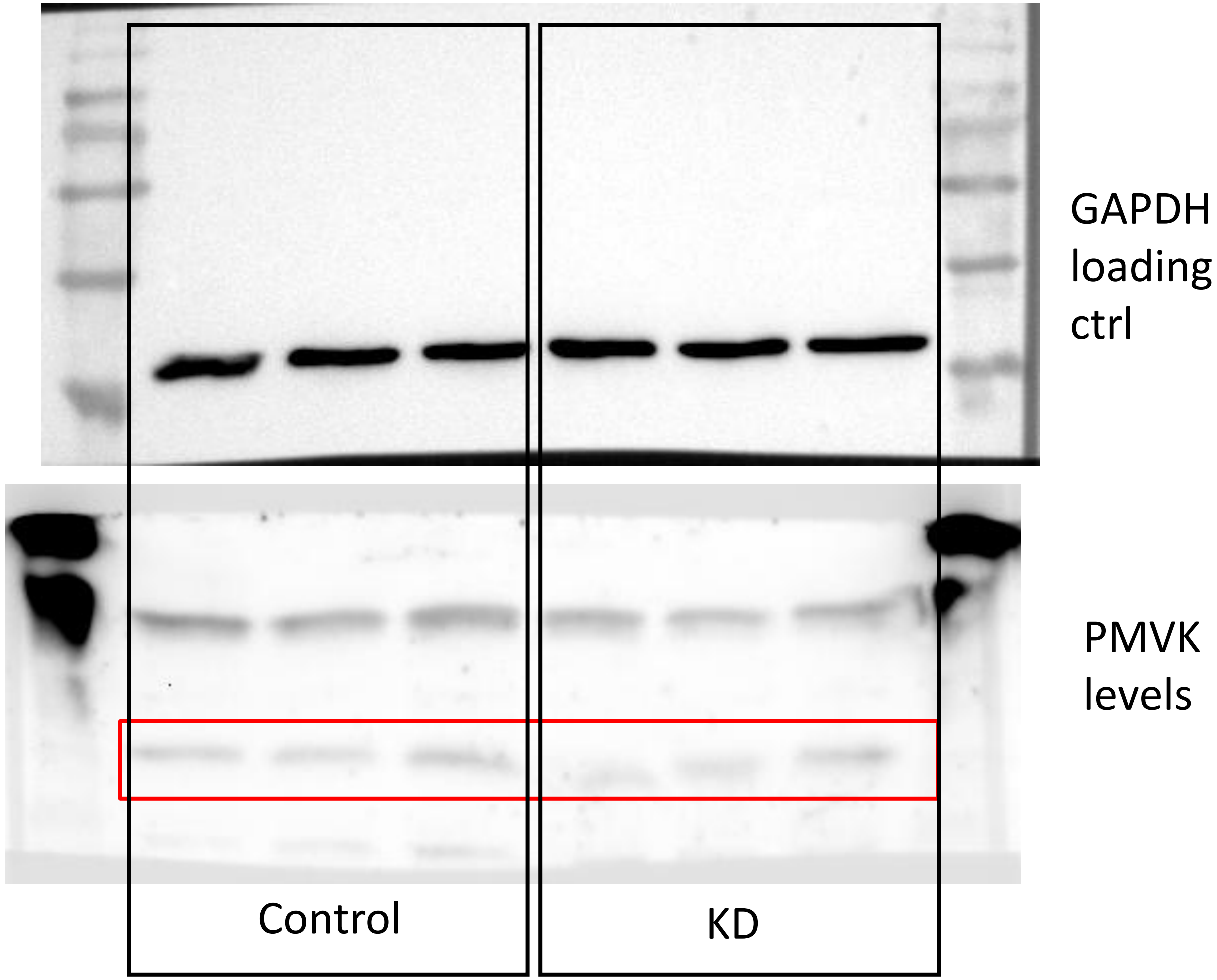

Western blot SiHa RNF20 KD lysates

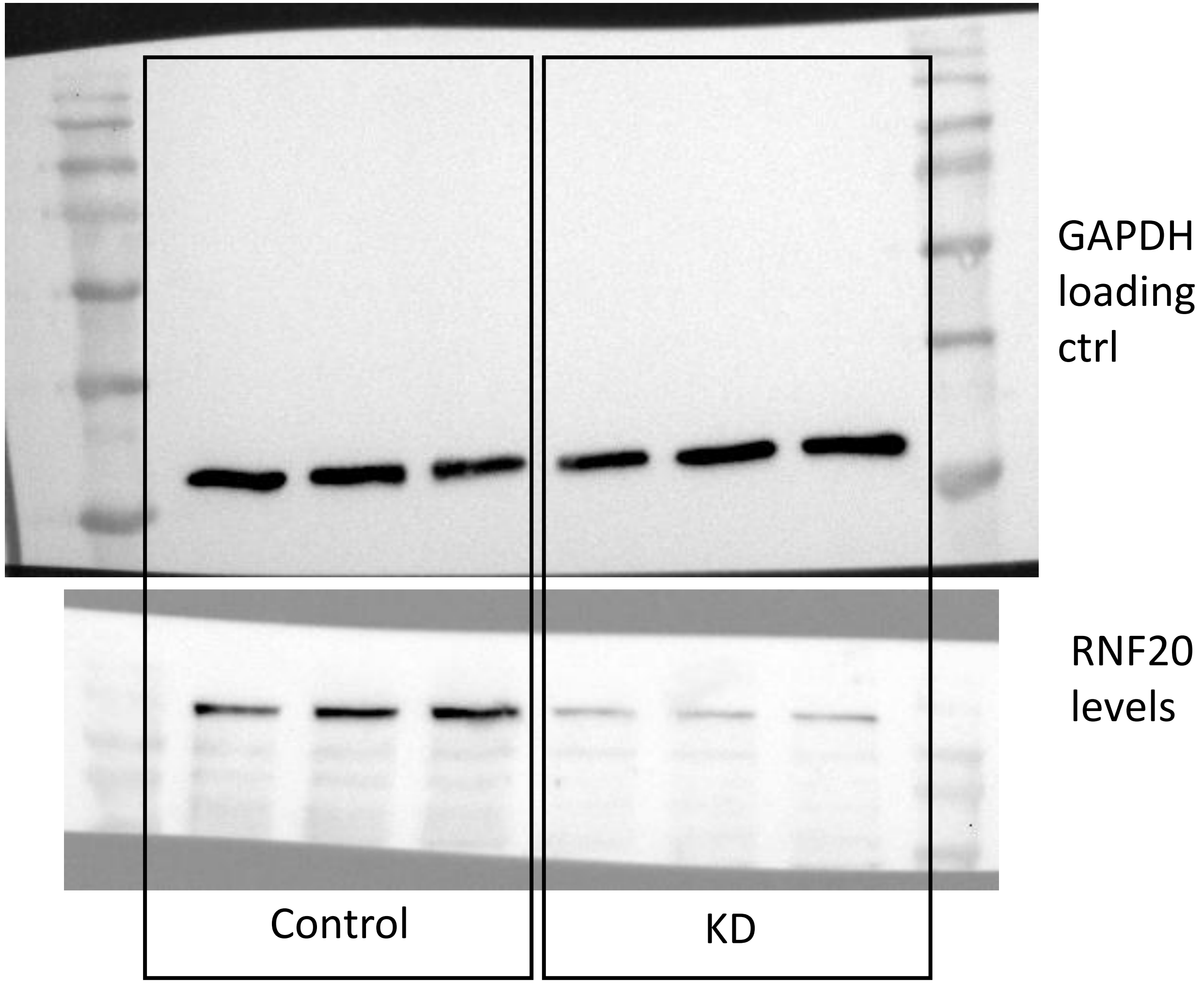

Western blot SiHa RNF40 KD lysates

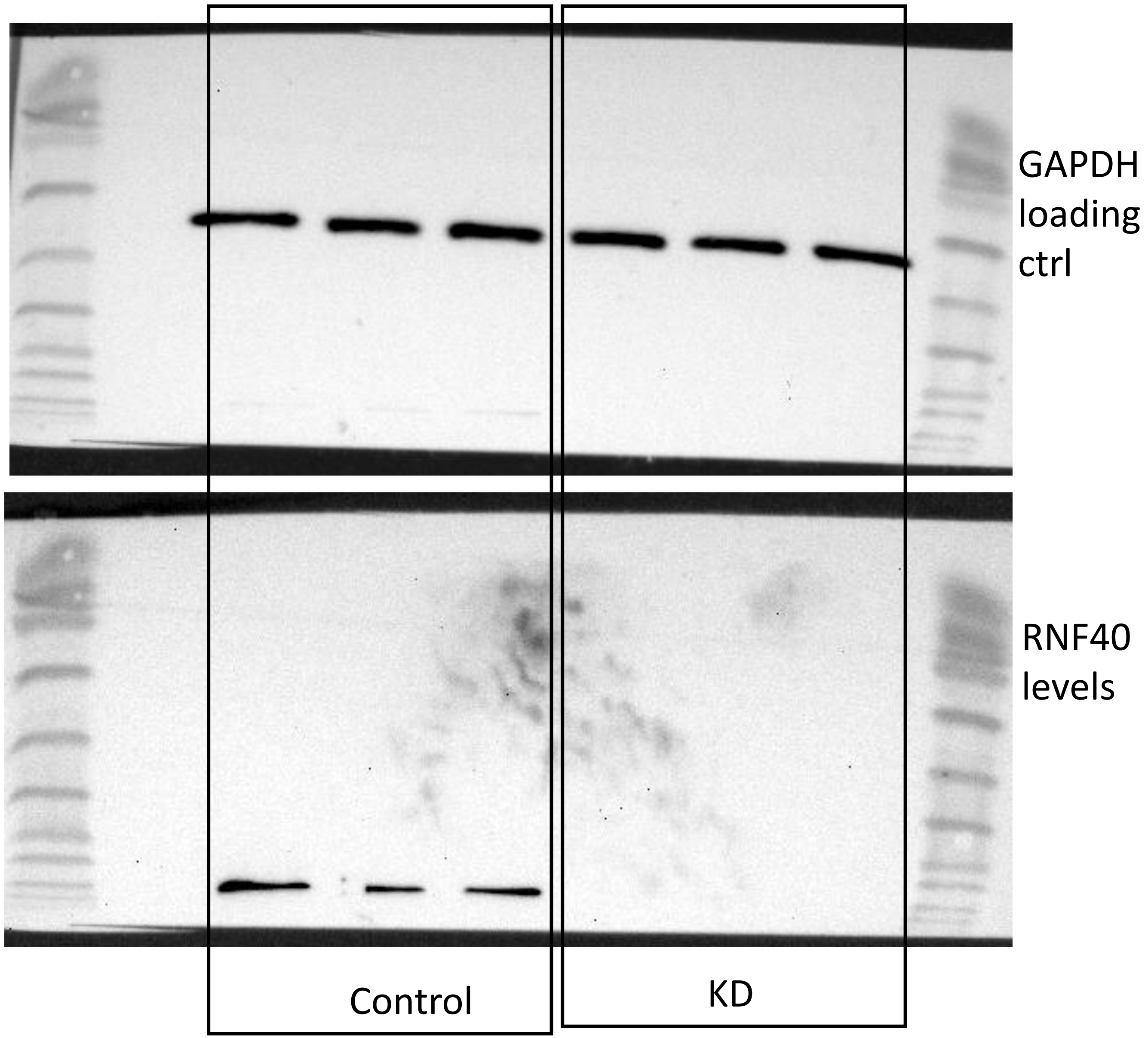

Western blot HeLa CDK9i lysates

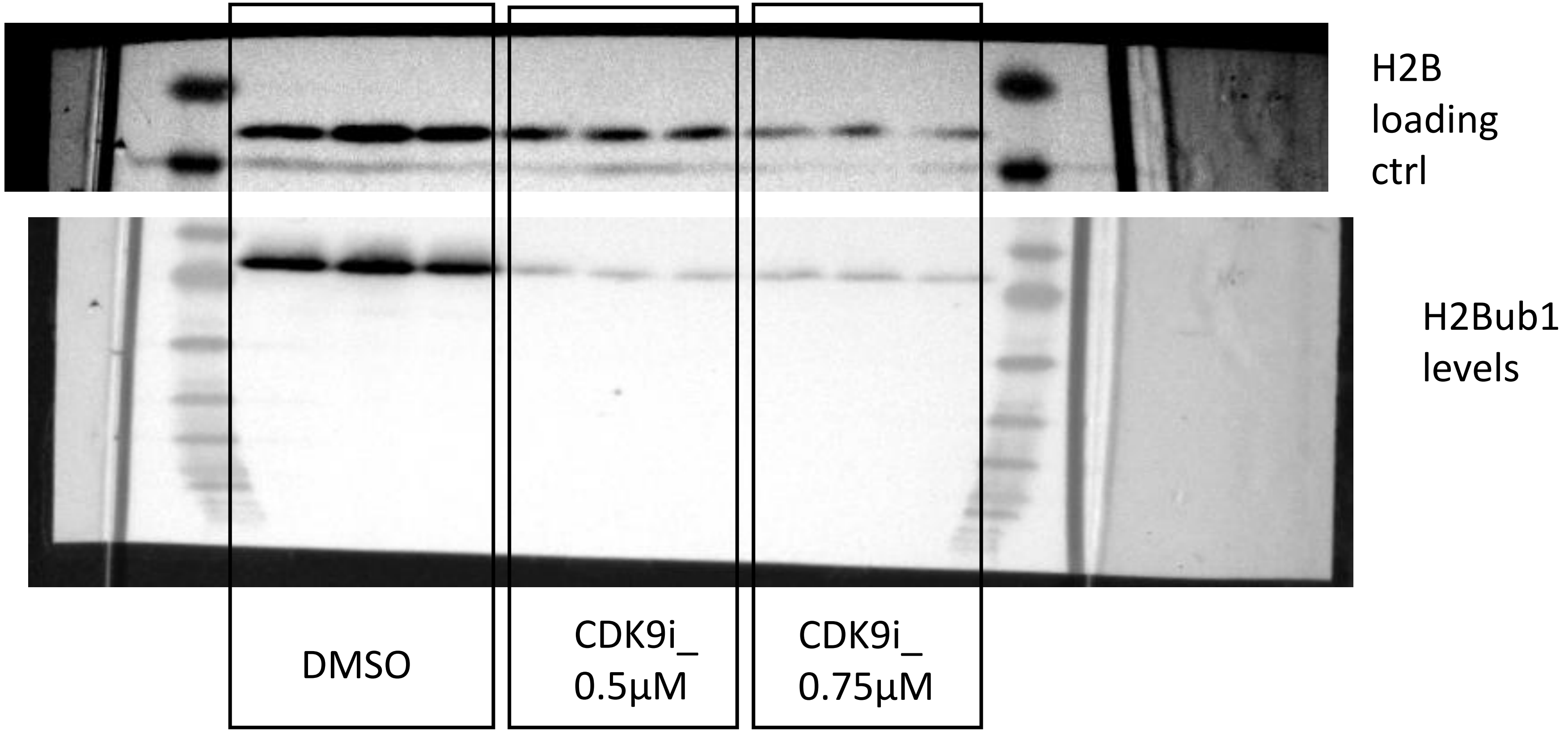

Western blot SiHa CDK9i lysates

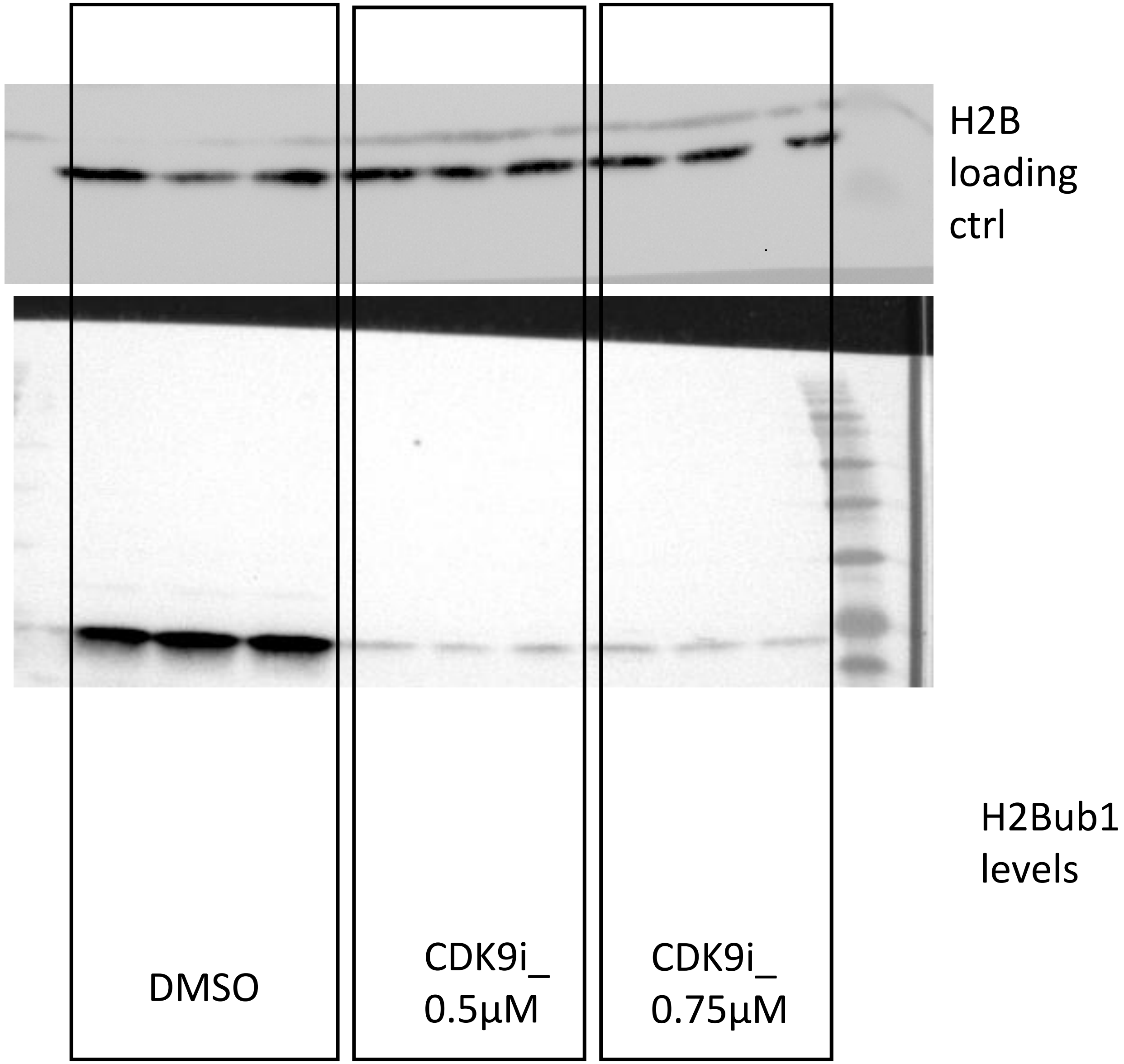

Supplement: Supplementary file 7 — Supplementary Material 7 [file 12964_2025_2279_MOESM7_ESM.pdf]
